# Amodiaquine Enhances Anti‐Melanoma Efficacy of Attenuated *Salmonella* via Targeting Glutathione Reductase in Neutrophils

**DOI:** 10.1002/advs.202515009

**Published:** 2026-01-26

**Authors:** Wanfa Dong, Chunyuan Zhao, Chengxi Li, Lin Weng, Zehan Ji, Peiqi Li, Jiqiang Lu, Danni Liu, Anni Yu, Tianyi Jiang, Shaokai Huang, Heng Liu, Xiao Chen, Zichun Hua

**Affiliations:** ^1^ School of Biopharmacy China Pharmaceutical University Nanjing P. R. China; ^2^ The State Key Laboratory of Pharmaceutical Biotechnology College of Life Sciences Nanjing University Nanjing P. R. China; ^3^ Yunnan Provincial Key Laboratory of Entomological Biopharmaceutical R&D College of Pharmacy Dali University Dali P. R. China; ^4^ Faculty of Pharmaceutical Sciences Xinxiang Medical University Xinxiang P. R. China; ^5^ Changzhou High‐Tech Research Institute of Nanjing University and Jiangsu Targetpharma Laboratories Inc. Changzhou P. R. China

**Keywords:** amodiaquine, glutathione reductase, neutrophils, ROS tolerance, target identification, VNP20009

## Abstract

Attenuated *Salmonella* VNP20009 (VNP) induces significant neutrophil recruitment in the tumor microenvironment (TME) during cancer therapy. However, the exact role of neutrophils in VNP‐mediated antitumor effects remains elusive. Here, we first identified the recruited neutrophils as predominantly N2 (pro‐tumor) subtype, which remarkably compromised VNP's antitumor efficacy. Therefore, we combined amodiaquine (AQ), known for its neutrophil‐inhibiting activity, with VNP treatment to enhance antitumor effects. The combination selectively inhibited TME neutrophils while maintaining favorable biosafety profiles. Employing chemical biology approaches, we identified glutathione reductase (GR) as the key target in neutrophils. Mechanistically, AQ binds to GR and compromises neutrophils' ROS tolerance, leading to selective elimination of neutrophils in the high‐ROS TME. A GR‐shRNA‐loaded VNP strain was further engineered and significantly potentiated VNP's antimelanoma effects. Our work not only advances the understanding of VNP‐immune‐tumor crosstalk but also provides a potential translational strategy that integrates drug repurposing with synthetic biology for developing microenvironment‐smart therapeutics.

## Graphical abstract

This study shows that *Salmonella* VNP20009 recruits pro‐tumor neutrophils that reduce its anticancer efficacy. Combining VNP with amodiaquine selectively eliminates these neutrophils by targeting glutathione reductase. A GR‐shRNA‐loaded VNP strain was further developed, demonstrating an innovative strategy integrating drug repurposing with synthetic biology for enhanced cancer therapy.

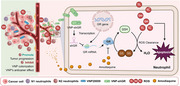

## 1 Introduction

The attenuated *Salmonella* strain VNP20009 (VNP) has emerged as a promising candidate for cancer therapy due to its inherent tumor‐targeting capability and selective colonization of hypoxic tumor regions [1]. Studies indicate that VNP not only directly eliminates tumors through cytotoxic factor release but also indirectly enhances antitumor immunity by remodeling the immune microenvironment and modulating various immune cell functions [2], including tumor‐associated macrophages (TAMs) and tumor‐infiltrating lymphocytes (TILs) [3, 4]. Despite these advantages, the clinical translation of VNP has been hampered by inconsistent therapeutic efficacy. This is primarily attributed to the immunosuppressive tumor microenvironment (TME), which was not addressed in the aforementioned clinical study [5, 6]. Significantly, the complex interactions between VNP and TME components—particularly immune cells—remain inadequately characterized. Elucidating these interactions is crucial for optimizing VNP's therapeutic potential, providing valuable insights to refine clinical protocols and broaden the applications of VNP‐based therapeutics.

Notably, the accumulation of neutrophils within tumors—a hallmark of many cancers—upon VNP treatment has been observed in multiple studies [7, 8]. Neutrophils play a crucial role in bacterial immunity; as the most abundant type of white blood cells in the human body, they serve as the first line of defense against bacterial infections in the innate immune system [9]. Therefore, neutrophils in the tumor microenvironment have the potential to inhibit VNP colonization at tumor sites. Beyond bacteria killing, recent studies indicate that neutrophils also participate in inflammatory responses and adaptive immunity [10, 11]. Currently, there is controversy regarding how neutrophils influence tumor progression, which may be related to the differentiation of tumor‐associated neutrophils (TANs) into pro‐tumor (N2) or antitumor (N1) phenotypes after infiltrating the TME in response to different stimuli [12, 13, 14]. At present, research on TANs primarily focuses on their effects in promoting tumor progression. For example, neutrophils can degrade the extracellular matrix by secreting proteases, creating conditions for tumor cell invasion [15]; N2‐type neutrophils can secrete various factors (such as VEGF, MMP‐9, IL‐8, etc.) that promote tumor angiogenesis and tumor cell proliferation [12]; neutrophils can help tumors evade immune surveillance by suppressing T cell and NK cell activity [16]. Additionally, tumors can induce neutrophils to form neutrophil extracellular traps (NETs), which can further promote tumor growth and metastasis [17]. Therefore, the enrichment of neutrophils in the TME may play an important regulatory role in tumor progression, and their impact on VNP therapy deserves further exploration.

Amodiaquine (AQ) is a classic quinoline‐based antimalarial agent that eliminates malarial parasites by inhibiting hemoglobin degradation in the parasite and interfering with DNA or RNA synthesis [18]. Clinical studies have demonstrated that neutropenia is a significant adverse reaction to AQ, typically occurring within 1–3 weeks after administration [19]. Mechanistic investigations have revealed that AQ causes neutrophil death through metabolism‐dependent cytotoxicity [20], suggesting its potential as a molecular tool for regulating neutrophil counts. In recent years, multiple studies have elucidated AQ's antitumor potential against various cancers, including breast cancer [21], cervical cancer [22], and lung cancer [23]. Notably, pharmacokinetic studies indicate that due to its lipophilic properties, AQ readily binds to lipids and substantially accumulates in adipose tissue upon entering the body. This preferential distribution in subcutaneous tissue allows AQ to form long‐term reservoirs, achieving slow release and prolonged retention [24]. Therefore, utilizing AQ to modulate neutrophil levels in the TME during VNP therapy for melanoma (primarily located in subcutaneous tissue) offers targeting advantages.

In this study, we initially demonstrated the therapeutic potential of VNP in melanoma treatment and observed a pronounced accumulation of neutrophils within the TME, particularly of the N2 phenotype. Subsequently, we confirmed at both cellular and animal levels that neutrophils promote tumor progression and inhibit VNP's therapeutic effects against melanoma. Considering AQ's neutrophil‐suppressive properties, antitumor effects, and targeting advantages in melanoma treatment, we utilized AQ in combination with VNP for melanoma therapy. Our findings revealed that AQ enhances VNP's therapeutic efficacy by selectively suppressing neutrophil levels in the TME. To understand the underlying mechanism, we designed and synthesized molecular probes of AQ and identified its protein targets in neutrophils through Activity‐based Protein Profiling (ABPP) coupled with quantitative proteomics. We elucidated that AQ decreases the reactive oxygen species (ROS) stress tolerance of neutrophils by inhibiting glutathione reductase (GR), leading to neutrophil apoptosis in the TME with elevated ROS levels. Based on these findings, we engineered a modified VNP strain carrying GR short hairpin RNA (VNP‐shGR) to induce neutrophil death by inhibiting GR levels of neutrophils in TME, thereby enhancing VNP's antitumor effects. This research contributes to our understanding of VNP's antitumor mechanisms, particularly its relationship with neutrophils in the TME, and provides important target information and lead compounds for the development of VNP combination therapies.

## 2 Results

### 2.1 VNP Exerts Anti‐Melanoma Activity and Recruits Neutrophils Within the TME

**FIGURE 1:**
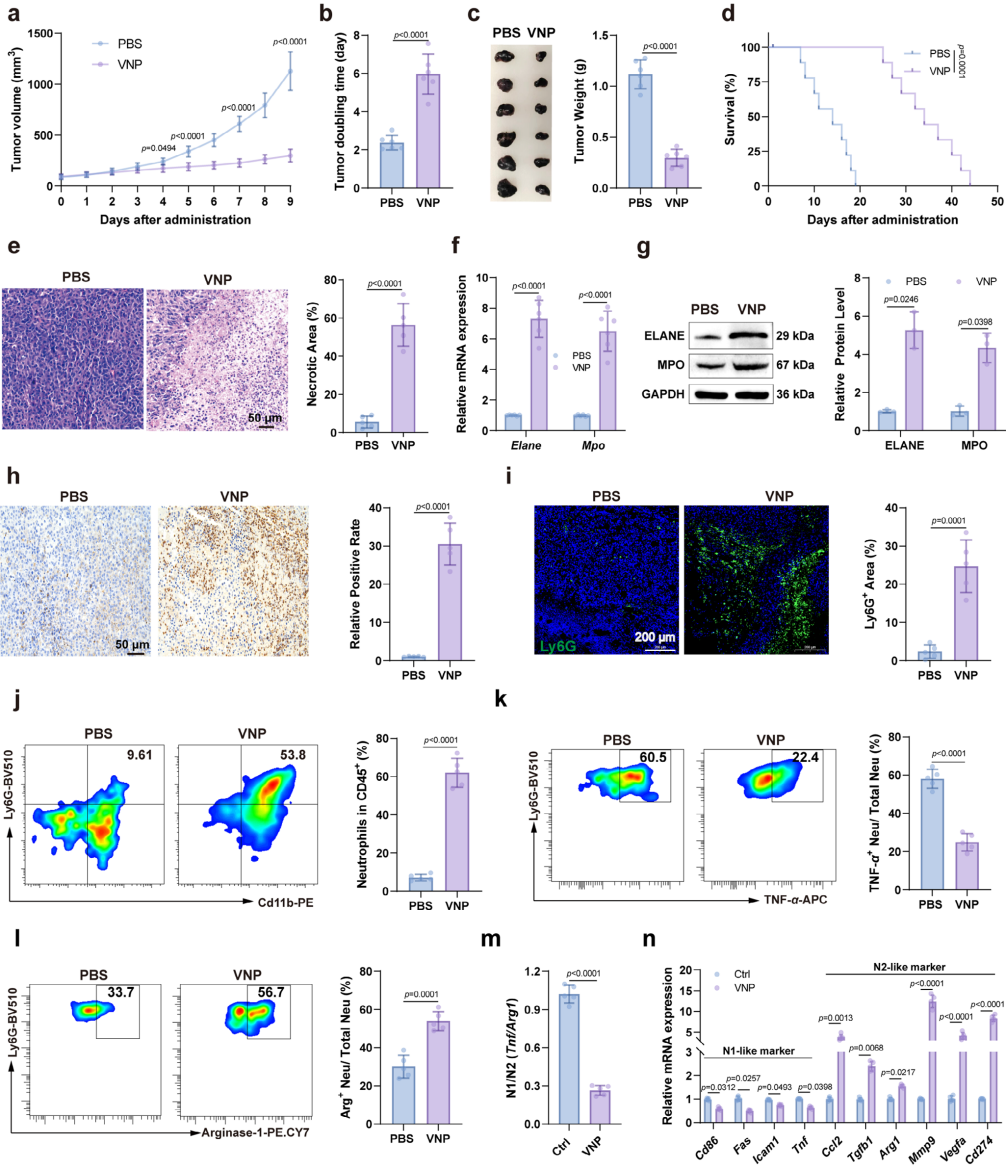
VNP treatment induces neutrophil recruitment in the tumor immune microenvironment. (a) Tumor growth curve after VNP treatment. (b) Tumor doubling time. (c) Tumor weight at the end point. (d) Survival curve of the model mice. (e) H&E staining of tumor tissues. Scale bar = 50 µm. (f) Relative mRNA expression of neutrophil marker genes Elane and MPO in tumor tissues. (g) Western blot analysis of Elane and MPO expression in tumor tissues. (h) MPO immunohistochemical staining of tumor tissues. Scale bar = 50 µm. (i) Ly6G immunofluorescence staining of tumor tissues. Scale bar = 200 µm. (j) Flow cytometry was employed to determine the proportion of neutrophils among immune cells in the tumor microenvironment following VNP therapy. (k,l) The proportions of N1 (k) or N2 (l) neutrophils among the total neutrophil population following VNP therapy. (m) The shift in N1/N2 neutrophil ratio in the total neutrophil population following VNP therapy. (n) Relative mRNA levels of N1 and N2 marker genes in total neutrophils within tumor tissues. Data represent the mean ± S.D. in a–c, f (*n* = 6), d (*n* = 9), e, h–m (*n* = 5), g (*n* = 3). Statistical significance was determined using an unpaired Student's *t*‐test in b,c, e, h–m. Two‐way ANOVA with Tukey's post hoc test was used in a, f,g, n. Log rank (Mantel–Cox) tests in d.

We first evaluated the therapeutic effects of VNP on the melanoma model mice. The results demonstrated that VNP significantly inhibited tumor growth in tumor‐bearing mice, as evidenced by slower tumor volume growth, prolonged tumor doubling time, and reduced tumor weight at the experimental endpoint (Figure 1a–c). Additionally, VNP treatment extended the survival rate of model mice (Figure 1d). Histological examination of tumor tissues by H&E staining revealed extensive necrotic areas in the VNP‐treated tumors (Figure 1e), consistent with VNP's inhibitory effect on tumor progression. However, VNP treatment also exhibited certain toxic side effects, primarily manifested as weight loss in the VNP treatment group and significant hepatosplenomegaly (Figure S1a–c). To explore the effects of VNP treatment on neutrophil recruitment in the TME, we utilized RT‐PCR and Western Blot to detect the expression of neutrophil marker proteins, Elastase (ELANE) and Myeloperoxidase (MPO), in tumor tissues from treated mice [25]. The results showed significantly elevated levels of ELANE and MPO in tumor tissues following VNP treatment (Figures 1f,g), indicating upregulation of neutrophils in tumor tissues. Using the neutrophil marker Ly6G and MPO antibody [26], we further confirmed VNP's effect on neutrophil recruitment through flow cytometry, immunohistochemistry, and immunofluorescence. These analyses revealed a notable increase in the proportion of neutrophils in the tumor immune microenvironment (TIM), consistent with our previous findings (Figure 1h–j). To determine the specific subtypes of neutrophils recruited by VNP, we analyzed the proportions of N1 (marked by *TNF‐α*) and N2 (marked by *Arginase‐1*) neutrophils within the total neutrophil population before and after VNP treatment using flow cytometry. The results demonstrated that VNP administration significantly reduced the proportion of N1 neutrophils (Figure 1k) while markedly increasing N2 neutrophils (Figure 1l). Consistently, RT‐PCR analysis revealed downregulation of N1 marker genes and upregulation of N2 marker genes in tumor‐associated neutrophils post‐VNP treatment (Figures 1m,n). These findings suggest that VNP preferentially recruits N2‐polarized neutrophils, which may promote tumor progression and attenuate the therapeutic efficacy of VNP. In conclusion, while VNP demonstrated significant anti‐melanoma efficacy, it concurrently induced neutrophil recruitment within the TME, with a predominant accumulation of the pro‐tumoral N2 neutrophil subtype.

### 2.2 Neutrophils Facilitate Tumor Progression and Attenuate the Anti‐Melanoma Efficacy of VNP

**FIGURE 2:**
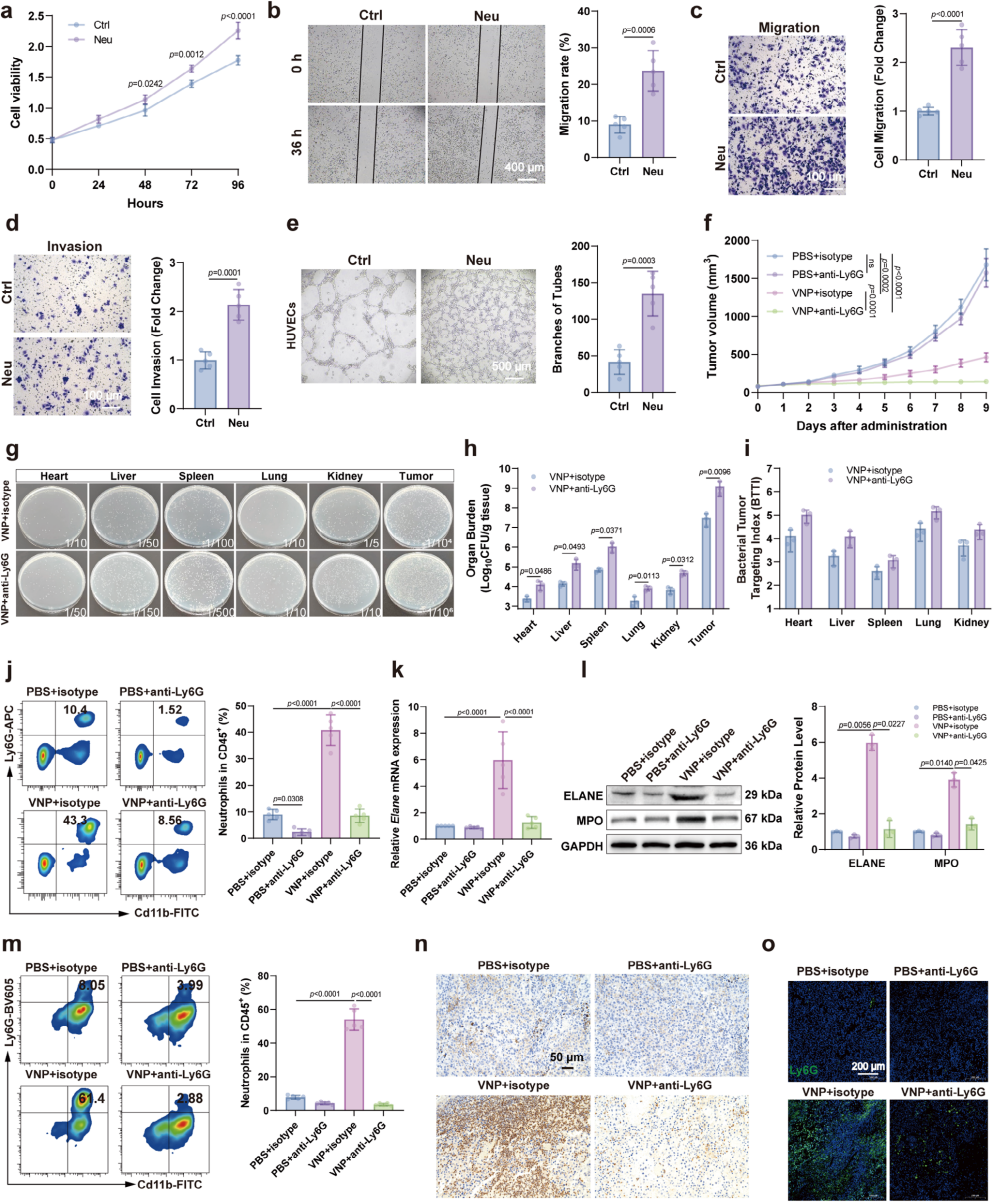
Eliminating tumor‐promoting neutrophils enhances the anti‐melanoma efficacy of VNP. (a) The effect of neutrophils on B16F10 cell viability. On day 9 after VNP therapy, subcutaneous B16F10 melanomas were collagenase‐digested to single‐cell suspensions; tumor‐associated neutrophils were magnetically sorted (#480058, BioLegend) and co‐cultured with B16F10 cells (1:1) in triple‐antibiotic (P/S/G) medium to prevent bacterial contamination. (b) Neutrophil‐mediated effects on B16F10 melanoma cell migration assessed by wound‐healing assay. Scale bar = 400 µm. (c) Neutrophil‐mediated effects on B16F10 melanoma cell migration assessed by trans‐well assay. Scale bar = 100 µm. (d) The effect of neutrophils on B16F10 cell invasion. Scale bar = 100 µm. (e) The effect of neutrophils on HUVECs tube formation. Scale bar = 500 µm. (f) Tumor growth curve after VNP or VNP combined with anti‐Ly6G treatment. (g) VNP plating on organ homogenates. The dilution ratio of the tissue homogenate is indicated in the corresponding panel. (h,i) Organ burden (h) and Bacterial Tumor Targeting Index (BTTI) (i) following VNP or VNP combined with anti‐Ly6G treatment. (j) Changes in the proportion of neutrophils among immune cells in the blood of mice following treatment with VNP combined with neutrophil‐neutralizing antibodies. (k) Relative mRNA expression levels of neutrophil marker genes in tumor tissues. (l) Western blot analysis of neutrophil marker protein expression in tumor tissues. (m) Flow cytometric analysis of the proportion of neutrophils in the tumor immune microenvironment. (n) MPO immunohistochemical staining of tumor tissues. Scale bar = 50 µm. (o) Ly6G immunofluorescence staining of tumor tissues. Scale bar = 200 µm. Data represent the mean ± S.D. in a–e, j,k, m (*n* = 5), f (*n* = 6), h,i, l (*n* = 3). Statistical significance was determined using an unpaired Student's *t*‐test in b–e. One‐way ANOVA with Tukey test was used in j, k, m. Two‐way ANOVA with Tukey's post hoc test was used in a, f, h,i, l.

Given the unclear role of neutrophils in melanoma progression, we evaluated their influence on melanoma cells at the cellular level. Our results revealed that VNP‐recruited neutrophils significantly promoted melanoma cell proliferation, migration, invasion, and HUVECs tubule formation (Figure 2a–e). ELISA results demonstrated that VNP significantly induced neutrophil secretion of pro‐tumorigenic factors, including IL‐8 (promoting tumor growth), MMP‐9 (promoting tumor metastasis), and VEGF‐*α* (mediating angiogenesis) (Figure S2a–c). Additionally, flow cytometry revealed that N2 neutrophils efficiently phagocytosed RFP‐labeled VNP (Figure S2d), indicating that N2 neutrophils promote tumor progression through dual mechanisms: secretion of pro‐tumorigenic factors and clearance of VNP. To explore the role of neutrophils during VNP treatment of melanoma, we used a neutrophil‐depleting antibody (anti‐Ly6G) in combination with VNP in a mouse melanoma model to decrease the neutrophil level in the TME. We first evaluated the efficacy of neutrophil depletion by detecting the neutrophil surface marker Ly6G in the blood. The results indicated that approximately 85% of neutrophils were depleted following Ly6G blockade (Figure S3a). Accompanied by the neutrophil clearance, the depleting antibody significantly enhanced the inhibitory effect of VNP on melanoma growth (Figure 2f; Figure S3b–d). However, the addition of anti‐Ly6G did not enhance VNP‐induced survival prolongation in tumor‐bearing mice; instead, it attenuated VNP's antitumor efficacy (Figure S3e), which may be attributed to the toxicity associated with anti‐Ly6G treatment. Considering neutrophils' phagocytic activity against bacteria, we examined how the depleting antibody affected bacterial tissue distribution. Results demonstrated that neutrophil depletion significantly increased VNP colonization in tumor tissue (Figure 2g–i) and enhanced VNP's tumor‐targeting capability (Figure 2j), which partially augmented VNP's antitumor efficacy. However, bacterial colonization in other organs simultaneously increased (Figure 2g–i), particularly in the liver and spleen, leading to certain toxic side effects manifested as decreased body weight and hepatosplenomegaly (Figure S4a–c). This may be related to the systemic reduction of neutrophils, including those in peripheral blood, liver, and spleen, caused by the depleting antibody (Figure 2j; Figure S4d,e). We further verified the effect of the depleting antibody on the level of neutrophils in tumor tissue. Results found that the depleting antibody significantly inhibited the expression of ELANE and MPO in tumor tissue of model mice (Figure 2k,l; Figure S5a) and reduced the proportion of neutrophils in the TIM (Figure 2m). It also downregulated neutrophil distribution in tumor tissue (Figure 2n,o; Figure S5b,c). These results indicate that neutrophil‐depleting antibodies enhance VNP's antitumor effect by inhibiting neutrophils in the TME.

### 2.3 AQ Enhances the Antitumor Efficacy of VNP by Selectively Eliminating Neutrophils in the TME

**FIGURE 3:**
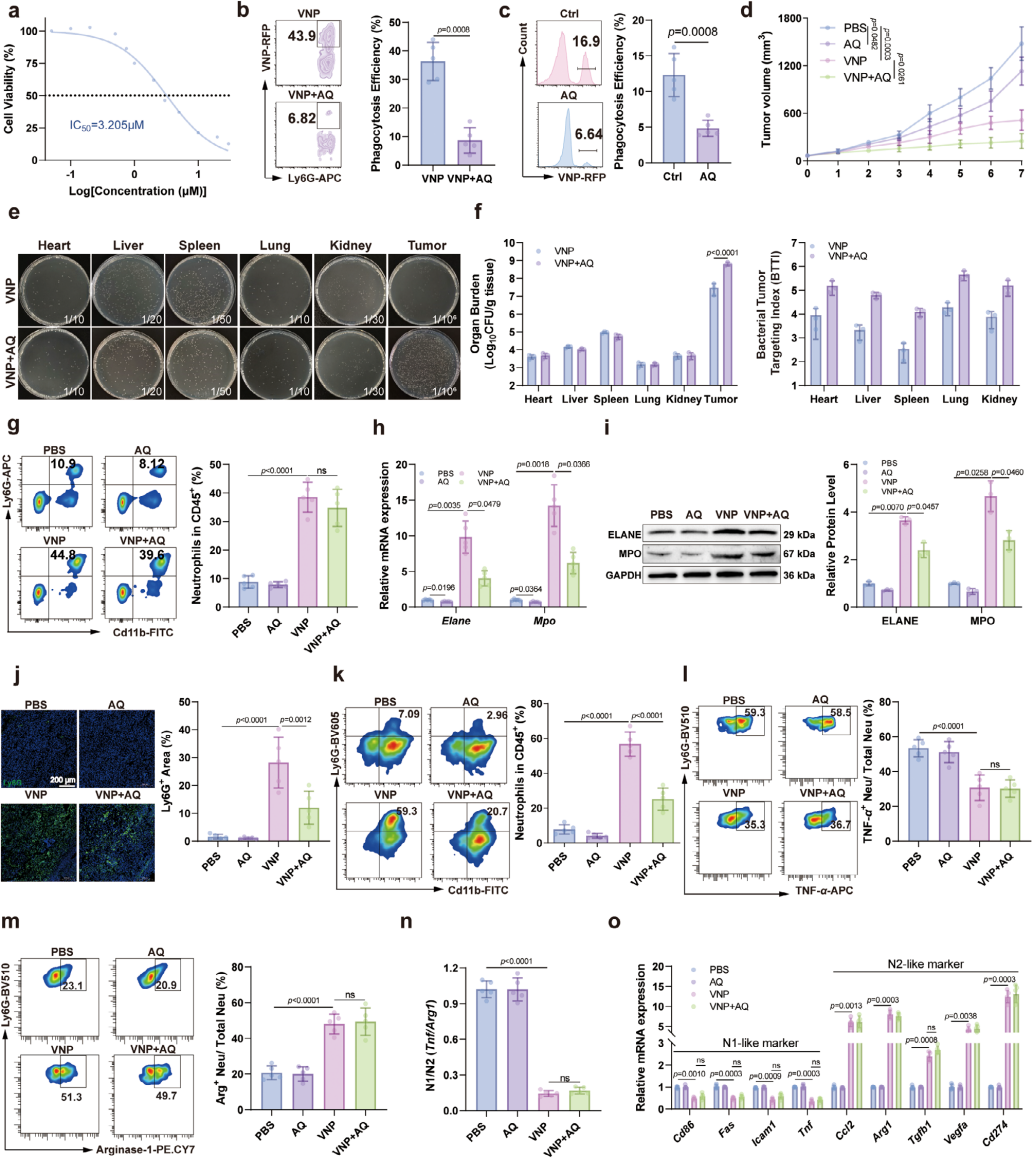
AQ enhances the antitumor efficacy of VNP by selectively eliminating neutrophils in TME. (a) IC_50_ values of AQ for neutrophils. (b,cEffects of AQ on neutrophil phagocytosis of VNP‐RFP in vitro and in vivo. (d) Tumor growth curve after VNP combined with AQ treatment. (e) Distribution of VNP in the mouse heart, liver, spleen, lung, kidney, and tumor after combined treatment with VNP and AQ. The dilution ratio of the tissue homogenate is indicated in the corresponding panel. (f) Organ burden and Bacterial Tumor Targeting Index (BTTI) following VNP combined with AQ treatment. (g) Changes in the proportion of neutrophils among immune cells in the blood of mice following treatment with VNP combined with AQ. (h) Relative mRNA expression of neutrophil marker genes in tumor tissues. (i) Western blot analysis of neutrophil protein expression in tumor tissues. (j) Ly6G immunofluorescence staining of tumor tissues. Scale bar = 200 µm. (k) Flow cytometry was employed to determine the proportion of neutrophils among immune cells in the TME following VNP combined with AQ therapy. (l,m) The proportions of N1 (l) or N2 (m) neutrophils among the total neutrophil population following VNP combined with AQ therapy. (n) The shift in N1/N2 neutrophil ratio in the total neutrophil population following VNP combined with AQ therapy. (o) Relative mRNA levels of N1 and N2 marker genes in total neutrophils within tumor tissues. Data represent the mean ± S.D. in a–c, g,h, j–o (*n* = 5), d (*n* = 7), e,f, i (*n* = 3). Statistical significance was determined using an unpaired Student's *t*‐test in b,c. One‐way ANOVA with Tukey test was used in g, j–n. Two‐way ANOVA with Tukey's post hoc test was used in d, f, h,i, o.

Compared to biologics and macromolecular drugs such as antibodies, small‐molecule inhibitors offer numerous advantages, including lower cost, relatively simpler production processes, better patient compliance through oral administration, more easily adjustable pharmacokinetic properties, shorter half‐lives, and relatively fewer side effects [27, 28]. Additionally, considering the unique advantages of drug repurposing in pharmaceutical development and AQ's potential inhibitory effects on neutrophils, we proposed using AQ in combination with VNP for melanoma treatment to achieve synergistic effects. Cytotoxicity experiments demonstrated that compared to normal cells, AQ exhibited stronger killing effects against neutrophils and B16F10 melanoma cells, with neutrophils showing greater sensitivity (Figure 3a; Figure S6a–c). In addition, AQ showed no significant cytotoxic effect on RAW264.7 macrophages (Figure S7). At low concentrations, AQ significantly inhibited neutrophil phagocytosis of VNP in vivo and in vitro (Figure 3b,c). In tumor‐bearing mouse models, AQ alone displayed weak antitumor effects but significantly enhanced VNP's inhibitory effects on melanoma growth (Figure 3d; Figure S8a,b), consistent with our hypothesis. Notably, the addition of AQ did not cause significant toxic side effects, as evidenced by no further decrease in body weight of model mice and no significant enlargement of the liver or spleen (Figure S8c,d). This may be related to the selective increase in VNP colonization and growth in tumor tissues after AQ treatment without affecting VNP levels in other organs (Figure 3e,f). To exclude the direct effects of AQ on VNP growth, in vivo results showed that AQ had no significant effect on VNP proliferation, colony formation, and morphology (Figure S9). We further found that the addition of AQ had no significant effect on peripheral blood neutrophil levels, nor on neutrophil levels in the liver or spleen (Figure 3g; Figure S10). RT‐PCR and Western Blot experiments on tumor tissues revealed that VNP significantly upregulated the transcription and expression levels of neutrophil‐specific genes Elane and Mpo, which were reversed by AQ (Figure 3h,i). Furthermore, AQ significantly inhibited the proportion of neutrophils among immune cells and the distribution of neutrophils in tumor tissues following VNP treatment (Figure 3j,k; Figure S11). These results indicate that AQ selectively reduced neutrophil levels in tumor tissues after VNP treatment. We further investigated whether AQ exhibits subtype‐specific cytotoxicity toward neutrophils in the TME. Both flow cytometry and RT‐PCR analyses demonstrated that AQ did not significantly alter the N1/N2 neutrophil ratio in the TME before and after VNP treatment (Figure 3l–o), indicating that AQ's neutrophil‐killing effect is not subtype‐selective. Given that N2‐polarized neutrophils predominated in the TME post‐VNP therapy, the AQ‐induced neutrophil depletion primarily affected the N2 subtype. This observation is consistent with the conclusion that AQ enhances the antitumor efficacy of VNP.

The biosafety of AQ combined with VNP therapy was also evaluated. Complete blood count (CBC) analysis results showed that VNP treatment significantly reduced lymphocyte and platelet levels, yet upregulated monocyte and neutrophil levels; AQ to some extent mitigated these VNP‐induced changes in blood cells (Figure S12). Histological results also indicated that the AQ and VNP combination had no significant toxicity to the heart, liver, spleen, lungs, or kidneys (Figure S13a). Additionally, liver and kidney function tests revealed that AQ alleviated the hepatic and renal toxicity caused by VNP (Figure S13b,c). Therefore, AQ enhances VNP's therapeutic effects against melanoma by selectively inhibiting tumor neutrophil levels while demonstrating good biosafety.

### 2.4 Target Identification of AQ in Neutrophils With Activity‐Based Protein Profiling (ABPP) Approach Combined With Quantitative Proteomics

**FIGURE 4:**
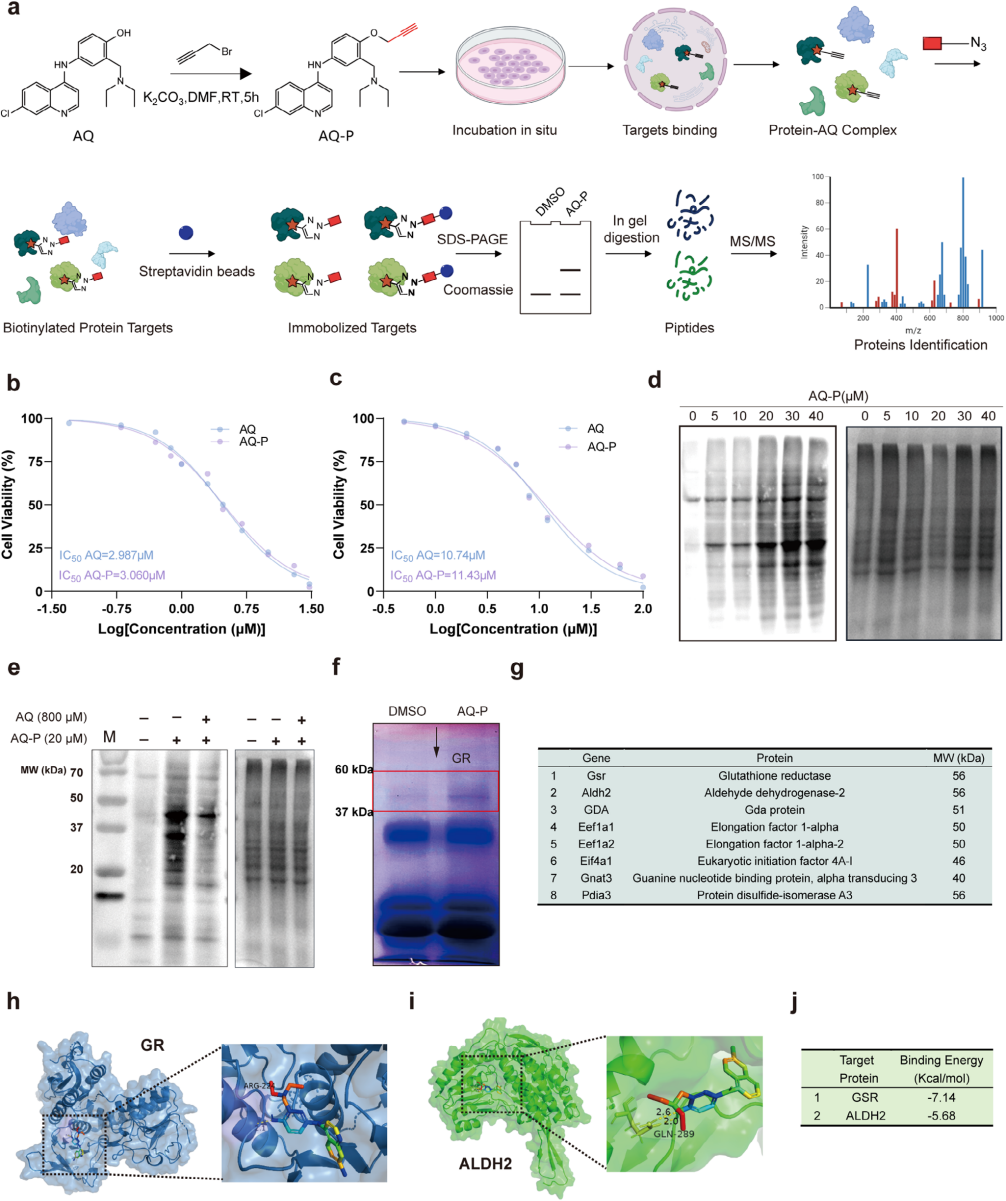
Targets identification with activity‐based protein profiling (ABPP). (a) Schematic illustration of the synthesis of AQ‐Probe and the workflow of ABPP to identify AQ protein targets in neutrophils. (b,c) IC_50_ values of AQ‐P for neutrophils and B16F10 cells. (d) Biotin‐modified proteins in neutrophils treated with different concentrations of AQ‐P. (e) The effect of high concentrations of AQ on the interaction between target proteins and AQ‐P. (f) Protein targets separated by SDS–PAGE and stained with Coomassie brilliant blue. (g) Candidate targets of AQ‐P in neutrophils. (h–j) Molecular docking predictions of binding sites and free energy changes for AQ interacting with GR and ALDH2. Data represent the mean ± S.D. in b,c (*n* = 5), d–f (*n* = 3).

Although previous studies have reported that AQ inhibits neutrophils through metabolism‐dependent pathways [20], the exact molecular mechanism remains unclear. Therefore, based on structure–activity relationship studies of AQ [29], we designed and synthesized an AQ probe (AQ‐P) (Figure S14) to identify its protein targets in neutrophils (Figure 4a). Briefly, tumor‐derived neutrophils were incubated with AQ‐P or DMSO to enable probe‐target binding. After cell lysis, Cu‐catalyzed azide–alkyne coupling reactions were performed to biotinylate the AQ‐P‐protein complexes via biotin–azide conjugation. The complexes were enriched using streptavidin beads, digested with trypsin, and analyzed by liquid chromatography‐mass spectrometry (LC‐MS) to identify AQ protein targets.

We first evaluated the pharmacological activity and specificity of AQ‐P to confirm that AQ‐P and AQ share the same protein targets. Cell viability experiments showed that AQ‐P can concentration‐dependently kill neutrophils and B16F10 cells, with IC_50_ values similar to those of AQ (Figure 4b,c), indicating that the pharmacological activity of AQ‐P is consistent with that of AQ. Target enrichment experiments revealed that as the concentration of AQ‐P increased, more proteins were biotinylated (Figure 4d). When the AQ‐P concentration reached 20 µm, the biotinylated proteins reached a peak level; therefore, we selected 20 µm as the experimental concentration of AQ‐P for subsequent target identification. Additionally, following established ABPP protocols where competitive inhibitors are typically used at 20–40 fold molar excess over the probe [30], high concentrations of AQ (800 µm) competitively inhibited the binding of AQ‐P to target proteins, suppressing their biotinylation modification (Figure 4e). These results fully confirmed the specificity of AQ‐P, namely that AQ‐P and AQ have consistent protein targets in neutrophils. We separated proteins enriched by DMSO or AQ‐P using SDS‐PAGE and, after Coomassie blue staining, found that AQ‐P successfully enriched some specifically bound proteins (Figure 4f). Mass spectrometry identification revealed 10 potential target proteins (Figure 4g). Considering that AQ functions through metabolic pathways, we selected two metabolism‐related enzymes from the target proteins, including glutathione reductase (GR) and aldehyde dehydrogenase 2 (ALDH2), as subjects for further investigation. Molecular docking simulations confirmed the direct interaction between GR or ALDH2 and AQ (Figure 4h,i), with binding affinities shown in Figure 4j. Together, these findings established GR and ALDH2 as bona fide molecular targets of AQ in neutrophils.

### 2.5 AQ directly binds to GR to inhibit its activity, thereby reducing the ROS stress tolerance of neutrophils

**FIGURE 5:**
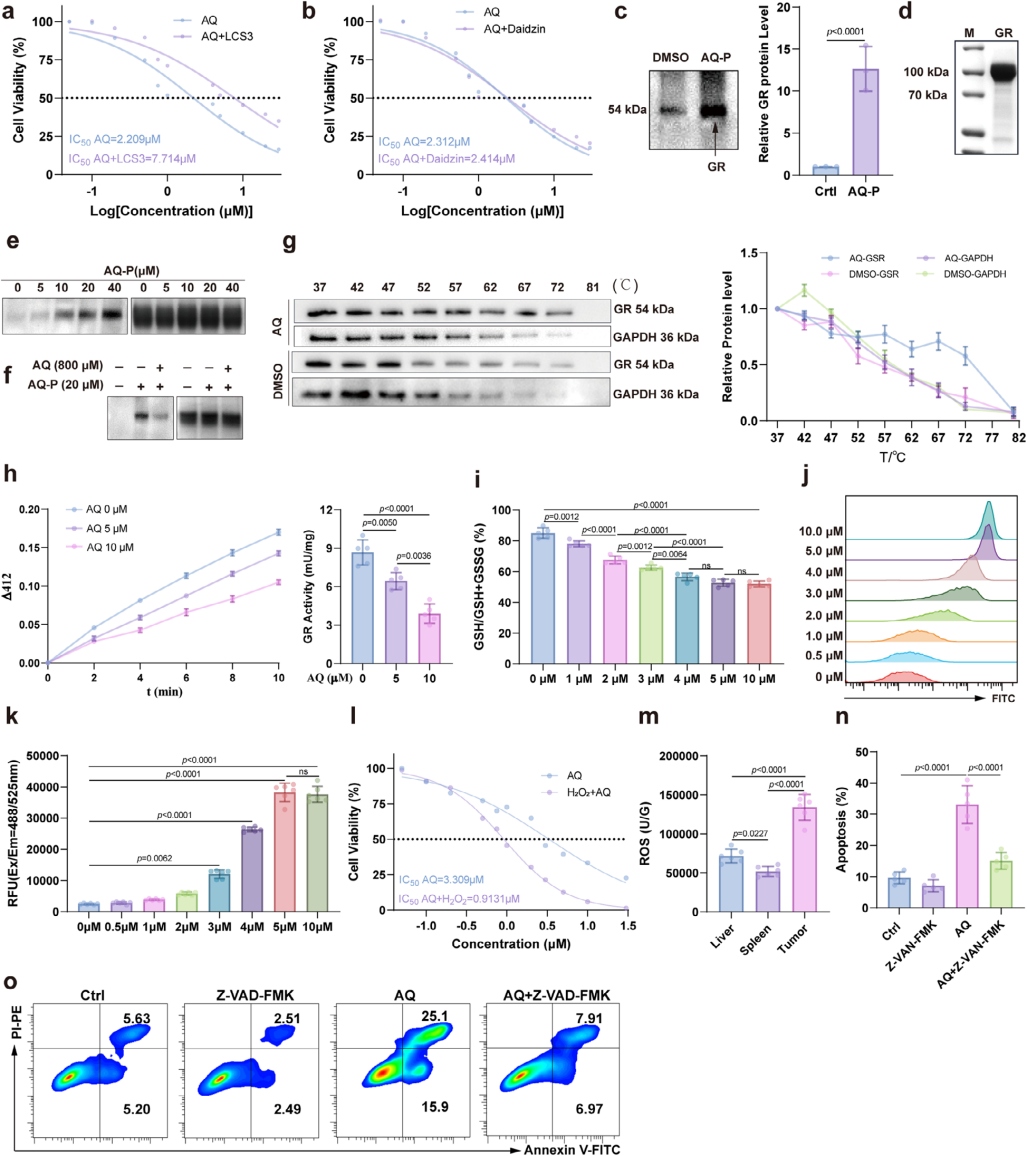
AQ induces neutrophil apoptosis by inhibiting GR activity and subsequently reducing ROS tolerance. (a,b) IC_50_ values of AQ in combination with LCS3 (a) or Daizdin (b) for neutrophils. Bone marrow–derived neutrophils were resuspended in antibiotic‐supplemented RPMI‐1640 (P/S/G) and seeded at 5 × 10^4^ cells per well of 96‐well plates. Cells were then co‐incubated for 24 h with the indicated concentrations of AQ in the presence of either LCS3 (5 µm) or daidzin (10 µm). Viability was subsequently assessed with the CCK‐8 assay. (c) Relative protein expression of GR in AQ‐P‐enriched proteins compared with DMSO. (d) Purified GR protein obtained through affinity chromatography. (e) Binding of purified GR with different concentrations of AQ‐P. (f) The effect of high concentrations of AQ on the interaction between purified GR and AQ‐P. (g) The effect of AQ on the thermal stability of GR in neutrophils. (h) Effects of AQ on GR activity in neutrophils. (i) the effect of AQ on GSH levels in neutrophils. (j,k) Flow cytometric (j) and microplate reader (k) analyses of the effects of AQ on ROS levels in neutrophils. (l) An ROS‐enriched environment enhances the susceptibility of neutrophils to AQ‐induced cytotoxicity. Murine bone‐marrow neutrophils (5 × 10^4^ cells/well) were plated in 96‐well plates containing antibiotic‐supplemented RPMI‐1640. Oxidative stress was induced with 20 µm H_2_O_2_; immediately afterward, serial dilutions of AQ were added to H_2_O_2_‐treated or control wells. After 24 h incubation at 37°C, 5 % CO_2_, viability was assessed by CCK‐8. (m) ROS levels in mouse liver, spleen, and melanoma tissues as determined by ELISA kit. (n,o) The impact of AQ on neutrophil apoptosis in a ROS‐rich environment. Data represent the mean ± S.D. in a,b, h,i, l–n (*n* = 5), k (n=6), c–g (*n* = 3). Statistical significance was determined using unpaired Student's *t*‐test in c. One‐way ANOVA with Tukey test was used in h,i, k, m,n.

To determine the key target from GR and ALDH2, we treated neutrophils with half‐inhibitory concentrations of GR or ALDH2 inhibitors (Figure S15a,b; Figure S16). Cell viability experiments demonstrated that the GR inhibitor LCS3 significantly reduced neutrophil sensitivity to AQ [31], while the ALDH2 inhibitor Daidzin showed no such effect (Figure 5a,b) [32], indicating that AQ's inhibition of neutrophil is mediated by GR. Western Blot experiments confirmed that AQ‐P enriched more GR compared to DMSO (Figure 5c). To verify the direct interaction between AQ and GR, we constructed a GR expression plasmid and expressed and purified GR protein (Figure 5d; Figure S17). Protein biotinylation detection showed that AQ‐P could bind to purified GR in a concentration‐dependent manner (Figure 5e), and high concentrations of AQ competitively inhibited the binding of AQ‐P to purified GR (Figure 5f). Since direct interactions between small molecules and proteins can enhance protein thermal stability [33], we also examined the effect of AQ on GR thermal stability. Results showed that AQ significantly enhanced GR thermal stability (Figure 5g). These findings indicate that AQ can directly bind to GR.

Since the GR activity inhibitor LCS3 can modulate AQ's cytotoxic effect on neutrophils, we hypothesized that the interaction between AQ and GR might lead to alterations in GR activity. Using a GR enzyme activity detection kit, we determined that AQ could inhibit GR catalytic activity in a concentration‐dependent manner (Figure 5h; Figure S18). GR catalyzes the conversion of oxidized glutathione (GSSG) to reduced glutathione (GSH), which is a central enzyme of cellular antioxidant defense [34]. Therefore, we further examined the effects of AQ on neutrophil GSH and ROS levels. We first assessed GR levels in neutrophils within the TME and found that, compared to liver and spleen tissues, neutrophils exhibited significantly higher GR expression, while GR levels in TME neutrophils showed no significant difference from those in the liver and spleen (Figure S19). AQ dose‐ and time‐dependently reduced intracellular GSH proportions (Figure 5i) and elevated ROS levels (Figure 5j,k; Figure S20), consistent with our hypothesis. After treating neutrophils with low concentrations of H_2_O_2_ (which had no significant effect on cell viability but could upregulate neutrophil ROS levels) (Figure S21), AQ's cytotoxic effect on neutrophils was enhanced (Figure 5l), indicating that AQ inhibits GR activity to reduce neutrophil tolerance to ROS. Considering the upregulation of ROS levels in the TME [35] (Figure 5m), we hypothesized that elevated ROS in tumors promotes AQ‐mediated killing of neutrophils in the TME, while due to lower ROS levels, AQ's cytotoxic effect on neutrophils in the liver and spleen was reduced. This finding is consistent with the animal‐level observation that AQ did not cause severe hepatosplenomegaly, and VNP colonization in the liver and spleen showed no increase. ROS scavenger NAC significantly reversed AQ‐induced neutrophil depletion in vivo, while NAC alone had no direct effect on neutrophils (Figure S22), confirming that ROS levels are a critical determinant of AQ's selective cytotoxicity toward neutrophils in the TME. Further studies revealed that under the ROS levels in the TME, AQ significantly induced neutrophil apoptosis, and this effect was reversed by the apoptosis inhibitor Z‐VAD‐FMK (Figure 5n,o; Figure S15c) [36]. Therefore, AQ selectively triggers neutrophil apoptosis in the TME by targeting GR to reduce ROS tolerance in neutrophils.

### 2.6 Engineered VNP Strain Exerts Enhanced Anti‐Melanoma Activity by Targeting GR in Neutrophils

In previous research, our group has successfully utilized VNP to establish an shRNA biological delivery system to inhibit the expression of target genes in host cells [37]. Therefore, we designed and engineered VNP loaded with GR shRNA (VNP‐shGR), aiming to enhance VNP's anti‐melanoma effects by suppressing neutrophils (Figure S23a). We first confirmed the inhibitory effect of shRNA on GR transcription and expression (Figure S23b,c), and selected shGR3, which exhibited the most potent inhibitory effect, for subsequent experiments. Considering the antibiotic‐free in vivo environment, we assessed the stability of shRNA plasmids carried by VNP. Results showed that shRNA plasmids remained stably retained in VNP under antibiotic‐free conditions (Figure S24). To further investigate the efficiency of plasmid nuclear transfer in neutrophils following VNP‐shGR infection, we examined the effects of VNP‐shGR on GR transcription and expression at different MOIs and time points. Results demonstrated that VNP‐shGR significantly inhibited GR transcription and expression in neutrophils in both a concentration‐dependent (MOI: 0–20) and time‐dependent (0–48 h) manner (Figure S25). Notably, VNP‐shGR showed no significant cytotoxic effect on RAW264.7 macrophages (Figure S26), confirming its selective action on neutrophils. Additionally, VNP‐shGR showed no significant differences from VNP in proliferation, colony formation ability, or morphology (Figure S27).

**FIGURE 6:**
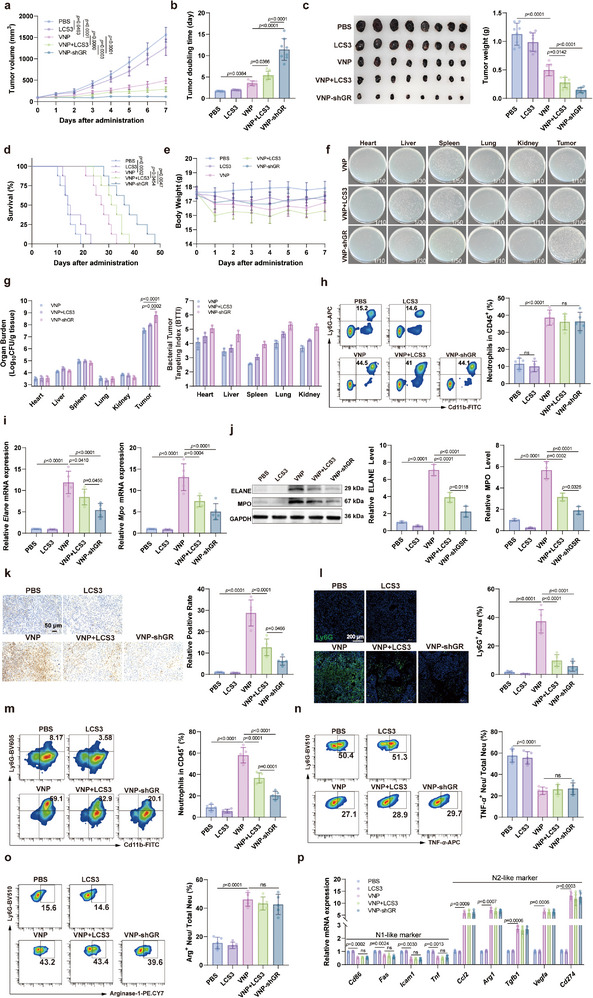
VNP‐shGR enhances the antitumor efficacy of VNP by targeting GR in neutrophils. (a) Tumor growth curve after engineered VNP and VNP combined with LCS3 treatment. (b) Tumor doubling time. (c) Tumor weight at the end point. (d) Survival curve. (e) Daily body weight changes of mice following VNP treatment. (f) VNP or VNP‐shGR plating on organ homogenates. The dilution ratio of the tissue homogenate is indicated in the corresponding panel. (g) Organ burden and Bacterial Tumor Targeting Index (BTTI) following engineered VNP and VNP combined with LCS3 treatment. (h) Changes in the proportion of neutrophils among immune cells in the blood of mice following treatment with VNP combined with LCS3 and VNP‐shGR. (i) Relative mRNA expression of neutrophil marker genes in tumor tissues. (j) Western blot analysis of neutrophil protein expression in tumor tissues. (k) MPO immunohistochemical staining of tumor tissues. Scale bar = 50 µm. (l) Ly6G immunofluorescence staining of tumor tissues. Scale bar = 200 µm. (m) Flow cytometry was employed to determine the proportion of neutrophils among immune cells in the TME in different groups. (n,o) The proportions of N1 (n) or N2 (o) neutrophils among the total neutrophil population. (p) Relative mRNA levels of N1 and N2 marker genes in total neutrophils within tumor tissues. Data represent the mean ± S.D. in a–c, e (*n* = 8), d (*n* = 10), g, j (*n* = 3), h–i, k–p (*n* = 5). Statistical significance was determined using One‐way ANOVA, and the Tukey test was used in b,c, h–o. Two‐way ANOVA with Tukey test was used in a, e, g, p. Log rank (Mantel–Cox) tests in d.

Using a melanoma model in mice, we compared the antitumor effects of VNP‐shGR and VNP. Results showed that compared to VNP alone, both VNP‐shGR and VNP combined with LCS3 demonstrated stronger inhibition of tumor growth, with VNP‐shGR showing the most pronounced effect (Figure 6a–c). Concurrently, VNP‐shGR and VNP combined with LCS3 significantly extended the survival time of tumor‐bearing mice (Figure 6d). Notably, compared to VNP alone, neither VNP‐shGR nor VNP combined with LCS3 caused severe adverse effects, including weight loss or hepatosplenomegaly (Figure 6e; Figure S28a,b). Furthermore, VNP‐shGR or VNP combined with LCS3 significantly enhanced bacterial colonization at tumor sites and promoted tumor targeting, without affecting bacterial colonization in the liver and spleen (Figure 6f,g), which is consistent with the therapeutic efficacy and safety evaluation results described above. Moreover, VNP‐shGR and VNP combined with LCS3 did not lead to significant decreases in neutrophils in peripheral blood, liver, or spleen (Figure 6h; Figure S28c,d); however, in tumor tissues, VNP‐shGR and VNP combined with LCS3 significantly inhibited VNP‐induced neutrophil recruitment in the TME (Figure 6i–m). Similarly, the reduction in neutrophils caused by VNP‐shGR or LCS3 was not subtype‐specific (Figure 6n–p; Figure S29). Organ toxicity evaluation results indicated that neither VNP‐shGR nor VNP combined with LCS3 exhibited obvious histological toxicity (Figure S30a), but both significantly improved the liver and kidney function damage caused by VNP, with VNP‐shGR showing more significant improvement (Figure S30b,c). Additionally, VNP, VNP combined with LCS3, and VNP‐shRNA moderately upregulated TNF‐*α*, IL‐6, and IL‐1*β* levels in serum (Figure S31), which remained far below the thresholds associated with systemic immune activation or cytokine storm. Therefore, VNP‐shGR enhances VNP's antitumor effect by inhibiting neutrophil GR levels to reduce their ROS tolerance, thereby selectively killing neutrophils in the TME.

## 3 Discussion

Bacterial therapies have garnered widespread attention across multiple diseases, yet emerging evidence reveals that microbial effects are highly context‐dependent and strain‐specific. For instance, gut microbiota dysbiosis can promote epithelial ovarian cancer progression by activating Hedgehog signaling via TLR4/NF‐κB pathways [38], while Micrococcus luteus isolated from osteoarthritic joints exacerbates cartilage degradation through TLR2/JNK/AP‐1 signaling [39]. Attenuated *Salmonella* VNP has demonstrated excellent antitumor efficacy in preclinical studies across various cancer types, primarily through its direct cytotoxic effects and by modulating the immune microenvironment [40, 41]. Notably, multiple studies have employed synthetic biology approaches to genetically engineer VNP for in situ immune modulation, thereby significantly enhancing antitumor immunity [6, 42, 43]. However, clinical trials have revealed that VNP monotherapy does not exhibit significant antitumor efficacy [5], hindering the broader clinical application and market approval of VNP‐based therapeutics. Therefore, engineering VNP based on its antitumor mechanisms to further enhance its antitumor activity while reducing side effects represents a key research focus. Currently, the mechanisms underlying VNP's antitumor effects remain incompletely understood, particularly regarding how immune cells within the TME interact with VNP during tumor killing processes. This knowledge gap has limited progress in VNP modification research. Consequently, elucidating how VNP therapy regulates the TIM and how key immune cells in the TME influence VNP therapy could guide the optimization of VNP strains, offering significant theoretical and clinical translational value.

Neutrophils, as key immune cells in defending against bacterial or fungal infections, have been found in multiple studies to be recruited into the TME following VNP treatment, leading to an increased proportion of neutrophils among immune cell populations and their enhanced distribution within the TME [37, 44]. In our study, we confirmed the elevation of neutrophils in the TME after VNP treatment. Therefore, we focused our research on the role of neutrophils in the TME regarding tumor progression and their impact on VNP's antitumor efficacy. However, current research yields conflicting findings regarding the role of neutrophils in tumor progression: N1‐type neutrophils exhibit antitumor effects, whereas N2‐type neutrophils promote tumor progression [45, 46]. For instance, Linde et al. found that activated neutrophils can promote the lytic action of neutrophils against tumor cells [47]; Finisguerra et al. discovered that neutrophils inhibit tumor growth and metastasis by inducing cancer cell death through the release of hepatocyte growth factor (HGF)/MET‐dependent nitric oxide [48]. More studies, however, suggest that neutrophils play a pro‐tumorigenic role. In a zebrafish model of RAS‐driven tumor formation, neutrophils enhanced cancer cell growth by releasing prostaglandin E^49^; in anaplastic thyroid cancer, changes in mitochondrial metabolism in tumor‐associated neutrophils allow them to maintain viability while releasing neutrophil extracellular traps (NETs), thereby promoting cancer cell proliferation [50]; the formation of NETs by neutrophils has been found in numerous studies to significantly promote cancer progression [51]. Therefore, we examined the subtype distribution of neutrophils recruited into the TME following VNP treatment and confirmed that VNP primarily increased the level of N2‐type (pro‐tumorigenic subtype) neutrophils in the TME. This finding suggests that VNP‐recruited neutrophils may promote tumor progression and attenuate the antitumor efficacy of VNP. Next, we assessed the impact of VNP‐recruited neutrophils on melanoma cells at the cellular level. Our results demonstrated that these neutrophils significantly enhanced melanoma cell proliferation, migration, invasion, and angiogenesis, consistent with our hypothesis. However, apart from neutrophils inhibiting VNP colonization in tumors through phagocytosis [52], we remain unclear about how neutrophils affect VNP efficacy. Using neutrophil‐depleting antibodies, we evaluated the inhibitory effect of VNP on melanoma after neutrophil depletion in vivo and determined that the recruitment of neutrophils to the TME weakened the therapeutic effect of VNP, while eliminating neutrophils could enhance VNP's antitumor action.

However, neutrophil‐depleting antibodies lack selectivity in their killing action against neutrophils. In addition to significantly suppressing neutrophils in the TME, they simultaneously downregulate neutrophil levels in peripheral blood, liver, and spleen, promoting bacterial colonization in these organs and resulting in hepatosplenomegaly, mouse weight loss, and other toxic side effects. Therefore, we considered using approved small‐molecule drugs to regulate neutrophil levels in the TME to enhance VNP's antitumor efficacy. AQ is primarily used clinically for the treatment and prevention of malaria. Due to increasing drug resistance, AQ monotherapy is no longer recommended and is mainly used in combination with other antimalarial drugs [53]. In recent years, AQ has been investigated for antitumor effects and autophagy regulation [54], though clinical translation remains at an early stage. Neutropenia is one of AQ's major side effects [55]. Considering AQ's relative biological safety and its potential targeting specificity for melanoma due to its unique metabolic process (accumulation in adipose tissue after entering the body with sustained release) [24], we proposed using AQ to inhibit neutrophils in the TME to enhance VNP's antitumor effect. Consistent with our expectations, results demonstrated that AQ significantly enhanced VNP's therapeutic effect against melanoma by inhibiting neutrophil levels in the TME. To our surprise, unlike neutrophil‐depleting antibodies, AQ exhibited selectivity in its killing action against TME neutrophils, showing no significant inhibitory effect on neutrophils in peripheral blood, liver, and spleen. This resulted in no increase in VNP colonization in the liver and spleen, and consequently, no apparent hepatosplenic toxicity. This selective effect might be related to AQ's tumor‐targeting properties and its mechanism of neutrophil inhibition. Therefore, elucidating the mechanism of AQ's selective killing of neutrophils in the TME could help identify targets for VNP combination therapy specifically targeting neutrophils in the TME.

To elucidate the molecular mechanism of AQ's inhibition of neutrophils, we synthesized AQ molecular probes and screened for AQ protein targets in neutrophils using chemical biology combined with quantitative proteomics approaches. Based on literature reports that AQ acts through a metabolism‐dependent pathway [20], we selected two metabolism‐related enzymes (GR and ALDH2) from among AQ's targets for further investigation. Under treatment with the GR inhibitor LCS3, AQ's relative killing effect on neutrophils was diminished, while the Aldh2 inhibitor showed no such effect, indicating that GR is a key target mediating AQ's neutrophil toxicity, with AQ exerting its inhibitory effect by modulating GR enzyme activity. An enzyme activity assay confirmed AQ's inhibitory effect on GR activity. Since GR primarily involves the conversion of reduced/oxidized glutathione in cells [20], we further examined AQ's impact on neutrophil GSH and ROS levels. Results showed that AQ significantly suppressed GSH levels while upregulating ROS levels. H_2_O_2_ stimulation experiments demonstrated that AQ reduced neutrophils' tolerance to ROS through GR inhibition, a finding that also resolved our questions regarding AQ's selective killing of neutrophils in the TME. Numerous studies have shown that tumor cells, due to metabolic abnormalities, produce higher levels of ROS, leading to abnormally elevated ROS levels in the TME [35, 56]. Our experimental results also confirmed this phenomenon: ROS levels in tumor tissues of model mice were significantly higher than those in the liver or spleen. Therefore, under the premise that AQ reduces neutrophil ROS tolerance, the high levels of ROS in the TME strongly induced neutrophil apoptosis, while neutrophils in the liver and spleen survived due to weaker ROS stimulation. Consequently, GR serves as a target for the selective killing of neutrophils in the TME. This discovery lays the theoretical foundation for the development of VNP combination drugs based on GR targeting.

Beyond its inherent antitumor effects, VNP serves as a delivery vector for therapeutic biologics (mRNA, shRNA, DNA) due to its tumor‐targeting properties [2, 57]. For example, Wu et al. employed VNP to deliver PD1 nanobody‐expressing plasmids, enabling targeted secretion within the TME^2^; our group engineered VNP carrying CCDC25 shRNA to suppress tumor metastasis via efficient intratumoral release [37]. Compared to conventional combinations (e.g., engineered bacteria plus small‐molecule inhibitors or antibodies like anti‐PD‐1), VNP‐mediated delivery offers key advantages: (1) Tumor selectivity—payloads accumulate exclusively in the TME via VNP's innate tropism, avoiding systemic exposure; (2) Self‐amplifying therapy—single‐dose VNP colonizes tumors and continuously produces therapeutics, eliminating repeated dosing; (3) Dynamic adaptation—living VNP vectors respond to evolving TME changes, unlike static drugs or antibodies.

Therefore, we constructed an engineered VNP strain carrying GR shRNA (VNP‐shGR) with the aim of selectively killing neutrophils in the TME by inhibiting their GR expression, thereby enhancing VNP's antitumor effect. We confirmed the engineered strain's ability to suppress GR expression levels at the cellular level, while also verifying that the bacterial modification did not affect the metabolic capabilities of the engineered strain, such as proliferation and colony formation. Currently, the primary mechanisms by which VNP‐shGR delivers shGR to TANs include three main pathways: 1) Type III Secretion System (T3SS)‐mediated delivery, where VNP's SPI‐1 and SPI‐2 encoded T3SS directly injects effector molecules and plasmid DNA into the host cell cytosol [58]; (2) phagocytosis and macropinocytosis‐mediated uptake, whereby neutrophils actively engulf bacteria through their antimicrobial functions or VNP manipulates the macro pinocytic pathway to enter cells, followed by intracellular cargo release; and (3) bacterial lysis and plasmid release, where VNP undergoes lysis within the harsh neutrophil intracellular environment characterized by reactive oxygen species and antimicrobial peptides, thereby releasing shGR‐encoding plasmids into the cytoplasm [59]. The relative contribution of each mechanism and which pathway predominates in the tumor microenvironment warrants further investigation.

Animal‐level experiments demonstrated that both the GR inhibitor LCS3 and the engineered strain VNP‐shGR significantly enhanced VNP's inhibitory effect on melanoma without exhibiting stronger toxic side effects, with VNP‐shGR showing superior results. This enhanced efficacy may be related to VNP‐shGR's dual targeting action: 1) decreased neutrophil ROS tolerance mediated by GR inhibition, and 2) accumulation of shGR in the TME due to VNP's inherent tumor‐targeting properties. These results further confirm that GR is an excellent target for the development of VNP combination therapies. Previous studies have established that GR is a critical enzyme for maintaining cellular redox homeostasis and is widely expressed in nearly all nucleated cells, with particularly high expression levels in hepatocytes, erythrocytes, and neurons [60]. Therefore, the potential risk of VNP‐shGR inhibiting GR activity in hepatocytes or erythrocytes warrants careful consideration. To address this concern, future iterations could incorporate an oxygen‐responsive suicide system into the engineered VNP strain, enabling programmed self‐destruction in normoxic non‐tumor tissues while preserving therapeutic function within the hypoxic TME. Such a safety switch would provide an additional layer of biological containment, ensuring that bacterial vectors are eliminated upon entering well‐oxygenated normal tissues, thereby minimizing off‐target effects on systemic GR activity.

Beyond neutrophils, other immune cells may also modulate VNP's antitumor efficacy through interactions with neutrophils, warranting further investigation in future studies. For instance, examining neutrophil‐T cell crosstalk through NET formation is critical, as neutrophils release PD‐L1‐decorated NETs that may directly induce CD8+ T cell exhaustion, thereby limiting therapeutic efficacy [61]. Additionally, elucidating how myeloid cells (macrophages, monocytes, and dendritic cells) influence neutrophil–bacteria interactions through LPS binding protein (LBP) secretion could reveal mechanisms by which these cells enhance neutrophil recognition and phagocytosis of Gram‐negative bacteria like VNP, ultimately reducing bacterial tumor colonization [62]. Furthermore, investigating neutrophil‐mediated suppression of NK cell cytotoxicity through arginase‐1 secretion and arginine depletion may uncover additional immunosuppressive mechanisms that bacterial therapy must overcome to achieve optimal therapeutic outcomes [63].

## 4 Conclusion

This study provides critical mechanistic insights into optimizing attenuated *Salmonella* VNP‐based cancer therapy by elucidating the immunosuppressive role of TANs in the TME. We demonstrated that VNP treatment preferentially recruits N2‐polarized neutrophils that promote tumor progression and attenuate therapeutic efficacy through dual mechanisms—inhibiting bacterial colonization via phagocytosis while simultaneously enhancing melanoma cell proliferation, migration, and angiogenesis. By identifying GR as a key therapeutic target through ABPP, we established that AQ selectively eliminates TANs in the high‐ROS TME by compromising their oxidative stress tolerance, thereby significantly enhancing VNP's anti‐melanoma efficacy without systemic toxicity. Furthermore, the engineered VNP‐shGR strain validated GR as an optimal target for combination therapy, achieving superior therapeutic outcomes compared to pharmacological GR inhibition alone. These findings not only advance our fundamental understanding of bacteria‐immune‐tumor interactions but also establish a potential translational framework that integrates drug repurposing with synthetic biology, offering a promising microenvironment‐smart therapeutic strategy for enhancing bacterial cancer therapy with improved efficacy and safety profiles.

## 5 Experimental Section

### 5.1 Animals and Cell Lines

Wild‐type female C57BL/6J mice (6–8 weeks old) were purchased from Huachuang Biotechnology Co., Ltd. (Nanjing, China). All animal experiments were conducted in accordance with protocols approved by the Animal Protection and Use Committee of China Pharmaceutical University (YSL‐20250425). The cell lines B16F10 (RRID: CVCL_XH27), AML12 (RRID: CVCL_0140), and NIH3T3 (RRID: CVCL_M025) were obtained from the American Type Culture Collection (ATCC) on May 18, 2023. Neutrophils were isolated from the femurs and tibias of the hind limbs of the mice, with the attached muscles and tissues removed. The bone marrow cavities were flushed with pre‐cooled PBS or complete culture medium using a syringe until the bone pieces turned pale. The flushed bone marrow cells were filtered through a 70‐µm cell strainer, lysed to remove red blood cells, and washed with pre‐cooled PBS. The resulting cells were then sorted using magnetic beads (#480058, BioLegend) and stored for subsequent use. The cells were cultured in RPMI 1640 medium (Thermo Fisher Scientific) supplemented with 10% fetal bovine serum (Gibco) at 37°C in a humidified atmosphere containing 5% CO_2_. B16F10 cells (5.0 × 10^5^ cells per mouse) were subcutaneously injected into the right flank of C57BL/6J mice. When the tumor volume reached approximately 80–160 mm^3^, VNP (1.0 × 10^6^ CFU per mouse) and VNP‐shGR strains (1.0×10^6^ CFU per mouse) were administered via a single intraperitoneal injection. Anti‐Ly6G antibody (BE0075, BioXCell) and anti‐mouse IgG2b antibody (BE0086, BioXCell) were administered at a dose of 50 µg per mouse daily for three days prior to bacterial treatment. AQ was administered intraperitoneally at a dose of 100 mg/kg per mouse (100 µL per mouse) daily, and LCS3 was administered intraperitoneally at a dose of 5 mg/kg per mouse (100 µL per mouse) daily. Tumor volume was measured daily using the formula V = 0.52×a×b^2^, where a represents the long diameter and b represents the short diameter. The tumor doubling time (TDT) was calculated using the formula TDT = t × log_2_/log (Vt/V_0_), where t represents the time interval between tumor assessments (in days), Vt represents the tumor volume at time t, and V_0_ represents the initial tumor volume. Mouse body weight was monitored daily. On the day of euthanasia, whole blood was collected for complete blood counts, and serum was analyzed for liver function markers (ALT, AST) and kidney function markers (urea nitrogen, creatinine). The heart, liver, spleen, lungs, and kidneys were weighed to calculate organ indices. Tissue sections were stained with hematoxylin and eosin (H&E) for pathological analysis, and tissue homogenates were prepared to examine bacterial distribution in the organs. Tumor sections were subjected to H&E staining, immunohistochemistry, and fluorescence immunostaining. Kaplan–Meier survival curves were plotted.

### 5.2 Competitive Binding Experiment of Amodiaquine with Amodiaquine Probe

Three experimental groups were conducted to evaluate the effects of varying concentrations of AQ and AQ‐P on cells. Group 1 served as a control with no AQ or AQ‐P added. Group 2 cells were treated with 800 µm AQ without AQ‐P. Group 3 cells were treated with both 800 µm AQ and 40 µm AQ‐P. After a 12‐h drug stimulation, cells were harvested, lysed by sonication, and centrifuged at 12 000 rpm for 20 min at 4°C. The supernatant was collected and sequentially treated with Biotin–azide (1 mm, 10 µL), TCEP (100 mm, 10 µL), TBTA (10 mm, 10 µL), and copper sulfate (100 mm, 10 µL), followed by vigorous shaking at room temperature for 4 h. The reaction mixtures were then transferred to ice‐cold acetone and precipitated overnight at −20°C. After centrifugation at 4°C and 12 000 rpm for 10 min, the supernatant was discarded. The protein pellets were resuspended in 150 µL loading buffer, dissolved, and boiled for 10 min at 100°C. Samples were subjected to SDS‐PAGE electrophoresis and subsequently transferred to a membrane at a constant current of 300 mA for 90 min. The membranes were then blocked with 5% skim milk for 1 h, followed by overnight incubation with HRP‐conjugated streptavidin. Afterward, the membranes were washed three times with PBST (10 min per wash) and finally visualized using a chemiluminescence detector.

### 5.3 Concentration Gradient Experiment of Amodiaquine Probe

Cells were co‐incubated with different concentrations of AQ‐P (0, 5, 10, 20, and 40 µm) for 24 h. The cells were then lysed by sonication, followed by centrifugation at 12 000 rpm for 20 min at 4°C. The supernatants were collected, and the following reagents were sequentially added: Biotin–azide (1 mm, 10 µL), TCEP (100 mm, 10 µL), TBTA (10 mm, 10 µL), and anhydrous copper sulfate (100 mm, 10 µL). The mixtures were vigorously shaken at room temperature for 4 h. The reaction solutions were transferred to ice‐cold acetone and precipitated overnight at −20°C. After centrifugation at 12 000 rpm for 10 min at 4°C, the supernatants were discarded. After the acetone had evaporated, 150 µL 1×Loading Buffer was added to each sample. The protein precipitates were dissolved and transferred to sterile centrifuge tubes. The samples were boiled at 100°C for 10 min and then subjected to SDS‐PAGE. After electrophoresis, the proteins were transferred to a PVDF membrane at a constant current of 300 mA for 90 min. The membrane was blocked with 5% skim milk for 1 h, followed by overnight incubation with HRP‐conjugated streptavidin. The membrane was washed three times with PBST (10 min per wash) and then visualized using a chemiluminescence imager.

### 5.4 Target Protein Enrichment by Activity‐Based Protein Profiling (ABPP)

Neutrophils were divided into two groups. The experimental group was treated with 40 µm AQ‐P, while the control group was incubated with an equal volume of DMSO for 24 h. The cells were lysed by sonication and centrifuged at 12 000 rpm for 20 min at 4°C. The supernatants were collected. Protein concentration was determined, and the protein content of both groups was strictly controlled at 4 mg. The following reagents were sequentially added: Biotin–azide (1 mm, 10 µL), TCEP (100 mm, 10 µL), TBTA (10 mm, 10 µL), and anhydrous copper sulfate (100 mm, 10 µL). The mixtures were vigorously shaken at room temperature for 4 h. The reaction solutions were transferred to ice‐cold acetone and precipitated overnight at −20°C. After centrifugation at 12 000 rpm for 10 min at 4°C, the supernatants were discarded. After the acetone had evaporated, the protein precipitates were dissolved in 1 mL of PBS containing 1% SDS. The volume was adjusted to 10 mL with PBS, and 90 µL of pre‐equilibrated streptavidin‐coated agarose beads was added. The samples were shaken at room temperature for 4 h. After washing, the beads were collected by centrifugation at 1000 g for 5 min and resuspended in 100 µL loading buffer. The samples were then separated by SDS‐PAGE and stained with Coomassie Brilliant Blue.

### 5.5 Cell Viability

B16F10, AML20, NIH3T3, and neutrophil cells were seeded at a density of 5 × 10^3^ cells per well in 96‐well plates and treated with varying concentrations of AQ, AQ‐Probe, LCS3, and Daidzin for 24 h. Additionally, B16F10 and neutrophil cells were co‐cultured at a 1:1 ratio for 24, 48, 72, and 96 h. After incubation, 10 µL of CCK‐8 reagent was added to each well and further incubated for 2 h. The absorbance was measured at 450 nm using a microplate reader.

### 5.6 Trans‐Well

Migration and invasion assays were conducted using 24‐well Trans‐well inserts with 8‐µm pores. B16F10 cells (5 × 10^3^) were suspended in serum‐free medium and seeded into the upper chamber, while the lower chamber was filled with medium containing 5 × 10^3^ neutrophils. After incubation for 24 h, cells that migrated or invaded through the membrane were fixed and stained, and then visualized under a microscope.

### 5.7 Wound Healing Assay

B16F10 cells (5 × 10^3^) were cultured in six‐well plates until they reached 90% confluence. A sterile 200‐µL pipette tip was used to create a scratch wound in the monolayer. The detached cells were removed by washing three times with PBS, followed by the addition of 2 mL of medium containing 5 × 10^3^ neutrophils for co‐culture for 24 h. The wound was imaged under a microscope after PBS was used to remove the co‐cultured neutrophils.

### 5.8 HUVECs Tube Formation Assay

Sixty microliters of Matrigel (BD Biosciences, USA) were plated in a pre‐cooled 96‐well plate and incubated at 37°C for 30 min. Human umbilical vein endothelial cells (HUVECs) were resuspended at a density of 3.0 × 10^4^ cells per 100 µL of DMEM medium, either alone or enriched with neutrophils at a 1:1 ratio. The HUVECs were then seeded into the 96‐well plate and cultured for 12 h in a 37°C, 5% CO_2_ incubator. Tube formation was observed and documented.

### 5.9 Apoptosis Assay

Bone‐marrow neutrophils were purified with a mouse bone‐marrow neutrophil isolation kit (#480058, BioLegend) and resuspended in antibiotic‐supplemented RPMI‐1640 (penicillin 100 U/mL, streptomycin 100 µg/mL). The cell density was adjusted to 5×10^5^ cells/mL, and 1 mL of the suspension was seeded into each well of a 12‐well plate. After 4 h of equilibration at 37°C / 5 % CO_2_, 20 µm H_2_O_2_ (Sigma) was added, and the cells were exposed for 12 h. The following treatment groups were then established: Ctrl, 3 µm AQ (MCE, USA), 40 µm Z‐VAD‐FMK (MCE, USA), and 3 µm AQ + 40 µm Z‐VAD‐FMK. Cells were cultured for an additional 24 h, gently detached by pipetting, and stained with an Annexin V‐FITC/PI apoptosis detection kit (Yeasen, China) according to the manufacturer's instructions. Apoptotic rates were quantified by flow cytometry on a FACSCanto II (BD Biosciences).

### 5.10 Quantitative PCR

Total RNA was extracted using Trizol reagent (Invitrogen). One microgram of RNA was reverse transcribed into cDNA using a cDNA synthesis kit (Vazyme, China). Relative mRNA levels were quantified using a one‐step RT‐PCR SYBR Green kit (Vazyme, China). Primers were synthesized by Sangon Biotech (Shanghai, China), and their sequences are provided in Table S1.

### 5.11 Western Blot

Cells were lysed on ice for 20 min using Sangon lysis buffer (China). The lysate was centrifuged at 12 000 rpm for 15 min, and the supernatant was collected. Protein concentration was determined using a BCA protein assay kit. Proteins were denatured, separated by SDS‐PAGE, and subsequently transferred to a PVDF membrane. The membrane was blocked with 5% skim milk and incubated with primary antibodies, followed by incubation with secondary antibodies. Target proteins were detected using Tanon ECL reagent. Secreted proteins in the bacterial culture medium were collected by TCA precipitation and processed as described for Western blot analysis. The antibodies used were: ELANE (Cat# MAB2204, RRID:AB_1674674), MPO (Cat# DS‐MB‐01961, RRID:AB_855278), and GR (Cat# 26‐852, RRID:AB_10905328), GAPDH (Cat# 2‐RGM2, RRID:AB_2721282).

### 5.12 Neutrophil Phagocytosis Assay

Neutrophils were isolated from mouse bone marrow and adjusted to a concentration of 1 × 10^6^ cells/mL. VNP‐RFP was added at a multiplicity of infection (MOI) of 100:1. Two groups were established: one treated with AQ at a concentration of 10 µm and another with the corresponding solvent. The cells were co‐cultured for 2 h. After co‐culture, cells were washed twice with PBS, centrifuged at 1200 rpm for 5 min each time, and the supernatant was discarded. The processed cells were resuspended in an appropriate volume of PBS and analyzed using a BD LSRFortessa flow cytometer to detect neutrophil RFP fluorescence. For in vivo assessment of neutrophil phagocytic capacity within the tumor immune microenvironment, mice were treated with either VNP‐RFP at a dose of 1.0 × 10^6^ CFU per mouse or VNP‐RFP at 1.0×10^6^ CFU plus AQ (100 mg/kg) per mouse. Tumor tissues were then harvested and analyzed by flow cytometry to evaluate RFP expression in neutrophils.

### 5.13 VNP Distribution In Vivo

Mice bearing subcutaneous melanoma tumors that had been treated with kanamycin‐resistant VNP bacteria were sacrificed, and their tumors and various organs were excised, weighed, and recorded. The tissues from each organ were minced and homogenized in an appropriate volume of sterile saline using a tissue grinder. A 100‐µL aliquot of the tissue homogenate was subjected to serial dilution. Subsequently, 100 µL of the diluted homogenate was evenly spread onto LB agar plates containing kanamycin. The plates were incubated at 37°C in an inverted position for 16 h to observe colony formation. The distribution of bacteria in different organs was analyzed to assess the organ targeting and enrichment of bacteria in the tumor tissue.

### 5.14 GR Protein Expression and Purification

The target gene GR was cloned into the pCold‐TF vector using a homologous recombination kit. The recombinant plasmid was transformed into BL21 cells. The cells were gently pipetted and incubated on ice for 30 min. The cells were then heat‐shocked in a water bath at 42°C for 90 s and immediately cooled on ice for 2 min. After adding 700 µL of LB (Luria‐Bertani) medium, the bacteria were cultured at 37°C with shaking at 200 rpm for 45 min. The bacterial suspension was then plated on solid LB agar containing ampicillin and incubated overnight at 37°C. Single colonies were selected and cultured in a larger volume of liquid LB medium. Protein expression was induced with 0.5 mm IPTG (Isopropyl‐*β*‐D‐thiogalactoside) at 15°C for 36 h. The bacteria were harvested by centrifugation at 4000 rpm for 20 min at 4°C, and the supernatant was discarded. The bacterial pellet was resuspended in Buffer A (20 mm Tris‐HCl, pH 8.5, 150 mm NaCl, 5% glycerol, 1 mm NADPH, 0.05 mg/mL FAD), lysed using a homogenizer, and centrifuged at 12 000 rpm for 20 min at 4°C. The supernatant was collected as the GR protein solution. The protein was purified using a Ni‐NTA affinity column. Impurities were washed off with Buffer A containing 20 mm imidazole, and the GR protein was eluted with Buffer A containing 50 mm imidazole. The purified protein was dialyzed to remove imidazole.

### 5.15 shRNA Design and Validation

Three highly efficient shRNA target sites were selected based on their interference efficiency. Oligonucleotides (Oligos) were designed with the stem‐loop sequence CTCGAG. The designed Oligos were cloned into the PRNA3‐SV40‐Neo‐U6 vector between the restriction sites BamHI and HindIII using standard cloning techniques. The ligation reaction was performed at 16°C for 1 h, followed by transformation into competent cells. The transformed cells were cultured overnight at 37°C. Single colonies were selected and sequenced to verify the correct insertion of the shRNA sequences. After confirming the correct sequences, the plasmids were extracted and transfected into B16F10 cells. Cells were collected, and RNA was extracted and reverse‐transcribed. The efficiency of the shRNA constructs was validated by RT‐PCR and western blotting. The shGR sequences were provided in Table S2.

### 5.16 Bacterial Growth Curve Determination

The OD_600_ of the VNP strains was adjusted to 1.0. Subsequently, 10 µL of the bacterial suspension was added to 0.5 mL of LB medium and incubated in a microplate reader (Synergy H1 BioTek) at 37°C for 24 h, with measurements of OD_600_ taken every 30 min for the generation of a growth curve.

### 5.17 Bacterial Strains and Plasmid Construction

VNP20009‐shGR, abbreviated as VNP‐shGR, was transformed with a PRNA3‐SV40‐Neo‐U6 vector expressing shGR. All plasmids were constructed using the ClonExpress II MultiS One Step Cloning Kit (Vazyme, C112/C113, Nanjing, China). The VNP strains were electro‐transformed with the plasmids as described previously.

### 5.18 Scanning Electron Microscopy (SEM)

VNP and VNP‐shGR were cultured to confluence on sterile coverslips and fixed with 2.5% glutaraldehyde in 0.1 m sodium cacodylate buffer (pH 7.4) for 2 h at room temperature. Bacteria were rinsed three times with PBS for 5 min each, post‐fixed with 1% OsO_4_ in PBS for 1 h at 4°C, and dehydrated through a graded ethanol or acetone series. The samples were then transferred to propylene oxide for 10 min. Coverslips were mounted onto SEM stubs using double‐sided adhesive tape, sputter‐coated with gold or gold‐palladium, and imaged in the SEM chamber at optimal pressure.

### 5.19 Identification of Target Proteins

The purified protein bands were excised from the stained gel using a sterile scalpel. Subsequently, the samples were desalted using Agilent Bond Elut OMIX Peptide‐based SPE and Millipore C18 ZipTip. The desalted samples were then analyzed using a TripleTOF 5600 system (AB SCIEX) in high‐resolution mode, with a resolution exceeding 30 000 and a mass range of 400–1250 m/z. The system was configured to accumulate signals for at least 250 ms per spectrum, selecting up to 20 precursors for fragmentation with charge states between 2 and 4. The dynamic exclusion time was set to 15 s. To further enhance sensitivity, a high‐sensitivity mode with a resolution of over 15 000 was employed. Protein identifications are listed in Table S3.

### 5.20 Glutathione Reductase Activity Assay

Neutrophils were divided into three groups and treated with 10 µm and 20 µm AQ or an equivalent volume of DMSO as a control. After a 12‐h incubation, cells were centrifuged, washed twice with PBS, and lysed in 200 µL of lysis buffer on ice for 30 min. The lysate was centrifuged at 12 000×g for 10 min at 4°C, and the supernatant was collected for GR activity measurement. The reaction mixture was prepared according to the manufacturer's instructions (S0055, Beyotime) and added to a 96‐well plate. After mixing, 6.6 µL of DTNB solution was added and mixed again. The plate reader was pre‐warmed to 25°C, and A412 was immediately measured and recorded as A412 (Time 0). A412 values were recorded every 10 min for 60 min. In a separate experiment, three samples were prepared with GR protein diluted to 43 nM and treated with 0, 10, and 20 µm AQ, incubated at room temperature for 1 h. The reaction mixture was prepared as described (S0055, Beyotime), and 6.6 µL of DTNB solution was added and mixed. The plate reader was pre‐warmed to 25°C, and A412 was immediately measured and recorded as A412 (Time 0). A412 values were recorded every 2 min for 10 min.

### 5.21 Measurement of GSH/GSSG

The intracellular level of GSH/GSSG was measured using GSH and GSSG Assay Kit (S0053; Beyotime). Neutrophils were isolated from mouse bone marrow using magnetic beads (#480058, BioLegend) and resuspended at a density of 1×10^6^ cells per well in RPMI 1640 medium supplemented with 10% FBS (Gibco). The cells were then seeded into six‐well plates. Each well was treated with AQ at a final concentration ranging from 0 to 10 µm for 1 h. Subsequently, the cells were centrifuged at 1000 rpm for 5 min and washed three times with PBS. According to the manufacturer's instructions, neutrophils were collected and lysed in a protein‐free solution, then frozen and thawed for three repetitively in liquid nitrogen and 37°C. Neutrophils were centrifuged at 10 000×g for 10 min at 4°C. The supernatant of the samples was extracted for the measurement of total GSH. The concentration of reduced glutathione (GSH) was calculated using the formula: GSH = Total Glutathione—(GSSG × 2).

### 5.22 Molecular Docking

The PDB file of Glutathione Reductase (GR) was obtained from the Protein Data Bank (PDB; http://www.rcsb.org/). The GR structure was processed using PyMOL to remove water molecules and other irrelevant small molecules. Duplicate structures were also removed. The GR protein was then imported into Auto‐Dock Tools 1.5.7, hydrogenated, and saved in PDBQT format. The AQ molecule, previously saved in MOL2 format, was also imported into Auto‐Dock Tools 1.5.7, hydrogenated, and saved in PDBQT format. Molecular docking was performed using Auto‐Dock Tools 1.5.7 with the Local Search 4.2 algorithm, and 50 docking runs were conducted. The docking results were evaluated based on binding energy and visualized using PyMOL.

### 5.23 Cellular Thermal Shift Assay

Proteins were extracted from neutrophils by repeated freeze‐thaw cycles in liquid nitrogen and supplemented with protease inhibitors. The extracted protein lysates were divided into two groups, one incubated with 20 µm AQ and the other with DMSO for 3 h. Each group was then divided into nine tubes and subjected to different temperatures in a PCR thermal cycler. The temperature gradient ranged from 37°C to 81°C, with an initial temperature of 37°C and increments of 5°C. After the reaction, the protein levels of GR were analyzed by Western Blotting.

### 5.24 Pull‐Down Assay

Two reaction groups were set up. One milliliter of purified GR protein solution (100 nM) was added to each group, followed by the addition of 40 µm AQ or an equivalent volume of DMSO. The mixtures were incubated at 4°C for 4 h. The following reagents were then added sequentially: Biotin–azide (1 mm, 10 µL), TCEP (100 mm, 10 µL), TBTA (10 mm, 10 µL), and anhydrous copper sulfate (100 mm, 10 µL). The mixtures were vigorously shaken at room temperature for 4 h. The reaction solutions were transferred to ice‐cold acetone and precipitated overnight at −20°C. The precipitates were collected by centrifugation at 12 000 rpm for 10 min at 4°C. After discarding the supernatant and allowing the acetone to evaporate, the protein precipitates were dissolved in 1 mL of PBS containing 1% SDS. The volume was adjusted to 10 mL with PBS, followed by the addition of 90 µL of pre‐equilibrated streptavidin‐coated agarose beads. After incubation with shaking at room temperature for 4 h, the beads were washed and pelleted by centrifugation (1000 × g, 5 min). The bead pellet was then resuspended in 100 µL of loading buffer, subjected to SDS‐PAGE, and analyzed by Western blotting for GR protein levels.

### 5.25 ROS measurement

Bone marrow‐derived neutrophils (1×10^6^ cells per well) were seeded into six‐well plates. For the experimental group, 1 µL of AQ was added to achieve a final concentration of 0–10 µm. The control group received an equivalent volume of solvent. After 8 h of treatment, cells were transferred to 1.5 mL centrifuge tubes and centrifuged at 1200 rpm for 5 min. The supernatant was discarded, and cells were washed three times with PBS. A staining solution was prepared by mixing 2 µL of DCFH‐DA (10 µm) with 3 mL of serum‐free medium, and the mixture was vortexed to ensure homogeneity while avoiding light. The supernatant from the centrifuged cells was discarded, and 1 mL of the prepared DCFH‐DA staining solution was added to resuspend the cells. The cells were then incubated in the dark for 30 min. After incubation, cells were washed twice with PBS, centrifuged at 1200 rpm for 5 min each time, and the supernatant was discarded. Finally, cells were resuspended in an appropriate volume of PBS and analyzed by flow cytometry to measure intracellular ROS levels. For the determination of ROS levels in mouse liver, spleen, and tumor tissues, the tissues were first weighed and placed into test tubes, followed by the addition of an appropriate volume of PBS. The test tubes were then subjected to tissue homogenization using a tissue grinder at 4°C, 60 Hz, for 45 s, with a pause of 15 s, and this cycle was repeated three times. After homogenization, the samples were centrifuged at 3000 rpm for 10 min at 4°C. The supernatants were collected and stored for subsequent procedures, which were carried out in accordance with the instructions provided in the ELISA kit (Zeweil, China).

### 5.26 Flow Cytometry

On Day 8 post‐treatment with VNP, samples of mouse melanoma tissue, liver, and spleen were collected and minced. The minced tissue was incubated with 1 mL of Collagenase IV (1 mg/mL) at 37°C for 30–60 min until complete digestion was achieved. The digested tissue was then filtered through a 70‐µm cell strainer to obtain a single‐cell suspension. The suspension was treated with red blood cell lysis buffer to remove erythrocytes, followed by washing with PBS. The cells were resuspended in PBS and adjusted to a concentration of 1×10^6^ cells/mL. A 100‐µL aliquot of the cell suspension was transferred to a flow cytometry tube and stained with 1 µL of anti‐CD45, anti‐CD11b, anti‐Ly6G, anti‐TNF‐*α*, and anti‐Arginase‐1 antibodies, respectively, in the dark for 30 min. The stained cells were washed twice with PBS, centrifuged at 1200 rpm for 5 min each time, and the supernatant was discarded. The stained cells were then resuspended in an appropriate volume of PBS and analyzed by flow cytometry (BD LSRFortessa) to detect cellular fluorescence. For the staining of the intracellular markers TNF‐α and Arginase‐1, the Cytofix/Cytoperm Fixation/Permeabilization Kit (BD, #554714) was employed, with the procedure carried out in strict accordance with the manufacturer's instructions. Data were analyzed using FlowJo software.

### 5.27 Synthesis of AQ‐P

AQ‐P was synthesized by alkylating the phenolic hydroxyl group of AQ with 5‐bromo‐1‐pentene in the presence of K_2_CO_3_ in DMF at room temperature for 5 h. The resulting allyl group‐containing AQ‐P was confirmed by LC‐MS, showing a molecular weight of 393.16 and a chemical formula of C_23_H_24_ClN_3_O.

### 5.28 Statistical Analysis

ImageJ software was used to quantify immunofluorescence area, fluorescence intensity, protein band density, and wound healing width. Data were analyzed using GraphPad Prism software v.9. Normality of the data was tested using the Shapiro–Wilk normality test and the Kolmogorov–Smirnov test. Experimental data were analyzed using a two‐tailed unpaired Student's *t* test, one‐ or two‐way ANOVA. Two‐sided Tukey's multiple comparison test was used for multiple comparisons. Statistical significance was defined as a probability value of 0.05 or less. Data are presented as the mean ± SD. Survival analysis was conducted using Kaplan–Meier survival curves, and differences between different mouse groups were assessed using log‐rank statistics.

## Funding

This work was supported by National Natural Science Foundation of China (32250016, 82130106, 82473954, 82303774); the Natural Science Foundation of Jiangsu Province (BK20243001, BG2024026, BY20241051, BE2023695, BK20230165, BK20230164, BK20231136, China); Yunnan Province Science and Technology Department (202505AF350090); Open Research Fund of Yunnan Characteristic Plant Extraction Laboratory (YKKF2023007, China), Jiangsu Province Youth Science and Technology Talent Support Program (JSTJ‐2024‐106, China), the Changzhou Municipal Department of Science and Technology (CE20246001, CJ20230017, CJ20235009, China), the Fundamental Research Funds for the Central Universities (0208‐14380191) and Jiangsu TargetPharma Laboratories Inc., China.

## Conflicts of Interest

The authors declare no conflicts of interest.

## Supporting information




**Supporting File**: advs73887‐sup‐0001‐SuppMat.docx.

## Data Availability

The data that support the findings of this study are available from the corresponding author upon reasonable request.
