# Supplementary material for: Amodiaquine Enhances Anti‐Melanoma Efficacy of Attenuated Salmonella via Targeting Glutathione Reductase in Neutrophils
Source: Adv Sci (Weinh). 2026 Jan 26;13(17):e15009. doi: 10.1002/advs.202515009 (PMC13042452; doi:10.1002/advs.202515009)
Supplement: Supplementary file 1 — Supporting File: advs73887‐sup‐0001‐SuppMat.docx. [file ADVS-13-e15009-s001.docx]

Supporting Information

**Amodiaquine enhances anti-melanoma efficacy of attenuated Salmonella via targeting glutathione reductase in neutrophils**

Wanfa Dong, Chunyuan Zhao, Chengxi Li, Lin Weng, Zehan Ji, Peiqi Li, Jiqiang Lu, Danni Liu, Anni Yu, Tianyi Jiang, Shaokai Huang, Heng Liu, Xiao Chen*, Zichun Hua*

**This file includes:**

Supplementary Materials and methods

Supplementary Figures. S1-32

Supplementary Tables. S1-3


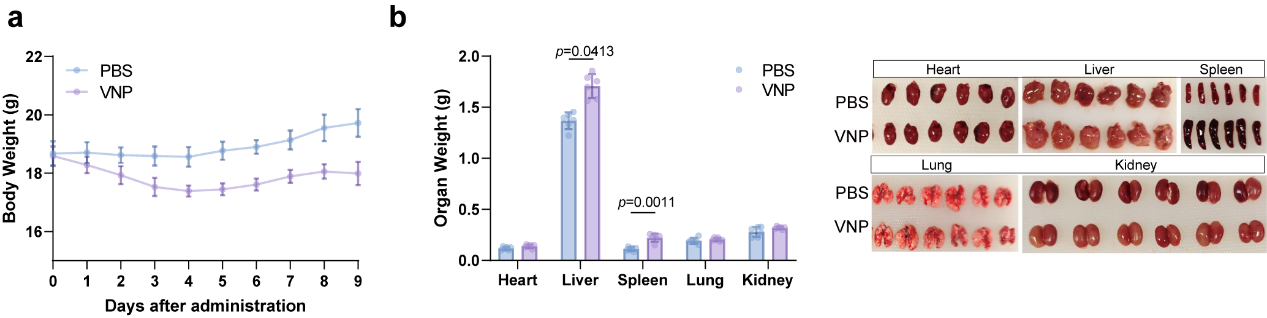


**Figure S1.** VNP demonstrated potent efficacy against subcutaneous melanoma. **a** Daily body weight changes of mice in each group following VNP treatment. **b** Organ weights. Data represent the mean ± S.D. in a-b (*n* = 6). Statistical significance was determined using two-way ANOVA with Tukey test in a-b.


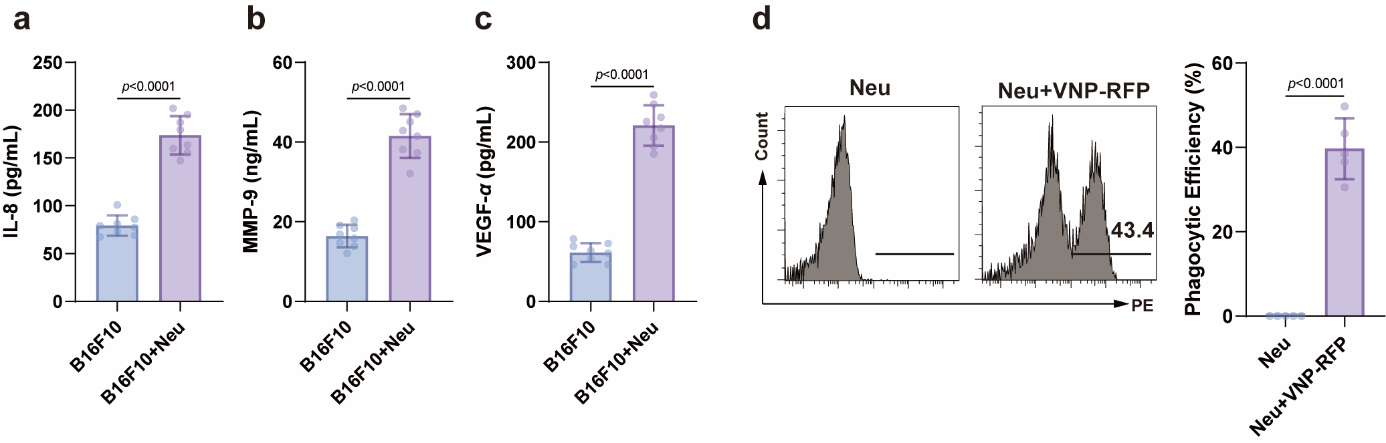


**Figure S2.** N2-polarized neutrophils isolated from VNP-treated tumours counteract VNP efficacy through secretion of pro-tumorigenic cytokines and direct phagocytic clearance. **a-c** ELISA detects the concentrations of IL-8 (**a**), MMP-9 (**b**) and VEGF-α (**c**) in 24 h co-culture supernatants of tumor-derived neutrophils after VNP treatment and B16F10 cells. **d** Flow-cytometric quantification of neutrophil uptake of VNP-RFP after co-culture for 6 h at a MOI 10. Data represent the mean ± S.D. in a-c (*n* = 8), d (*n* = 5). Statistical significance was determined using unpaired Student’s *t*-test in a-d.


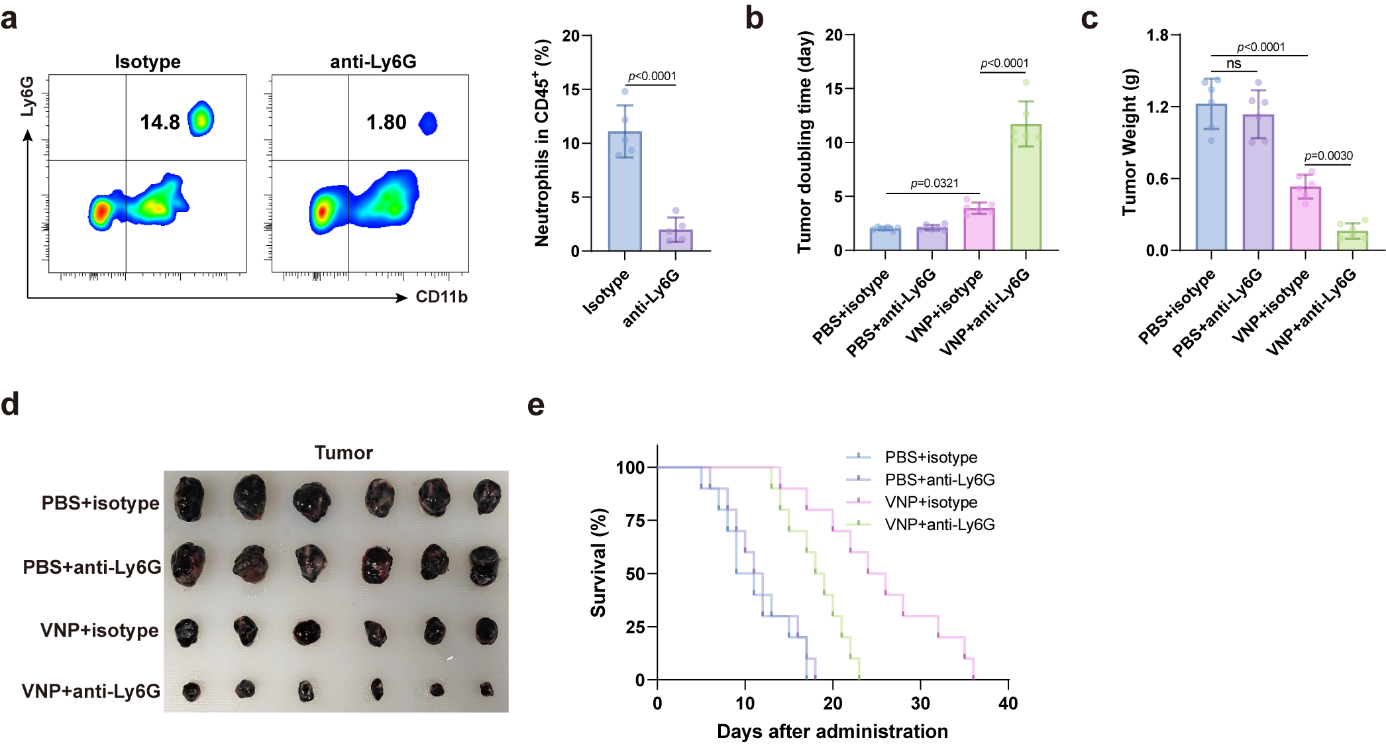


**Figure S3.** VNP combined with neutrophil depletion antibody enhanced antitumor efficacy. **a** The efficiency of neutrophil neutralizing antibodies in eliminating neutrophils from the bloodstream. **b** Tumor doubling time. **c** Tumor weight. **d** Tumor photographs at the end point. **e** Kaplan–Meier survival analysis of tumour-bearing mice receiving the indicated therapeutic regimens. Data represent the mean ± S.D. in a (*n* = 5), b-c (*n* = 6), e (*n* = 10). Statistical significance was determined using unpaired Student’s *t*-test in a. One-way ANOVA with Tukey test was used in b-c. Log rank (Mantel–Cox) tests in e.


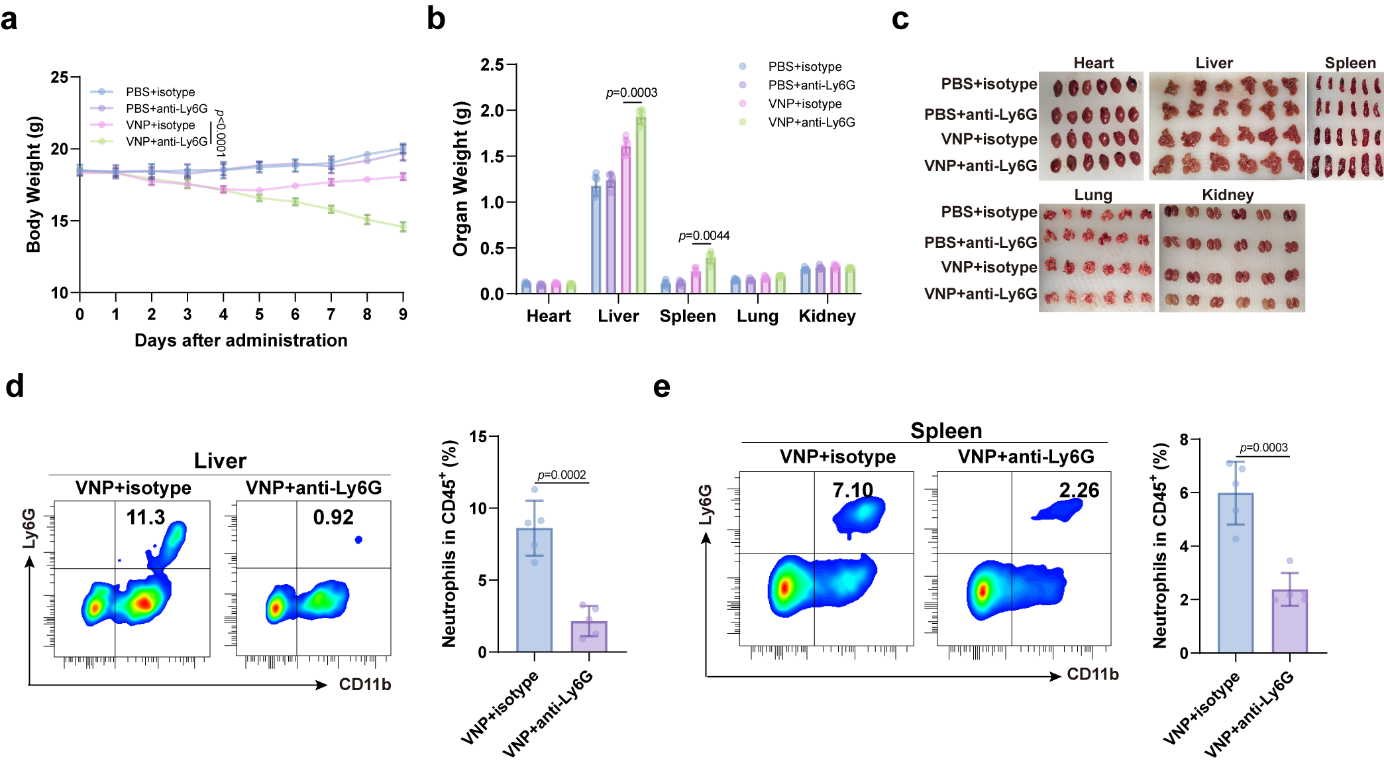


**Figure S4.** VNP combined with neutrophil depletion antibody exacerbated hepatosplenomegaly in mice. **a** Daily body weight changes of mice in each group following VNP treatment. **b** Organ weights. **c** Organ photographs. **d**-**e** The proportion of neutrophils among immune cells in the liver (**d**) and spleen (**e**) of mice after combined treatment with VNP and neutrophil neutralizing antibodies. Data represent the mean ± S.D. in a-c (*n* = 6), d-e (*n* = 5). Statistical significance was determined using unpaired Student’s *t*-test in d-e. Two-way ANOVA with Tukey test was used in a-b.


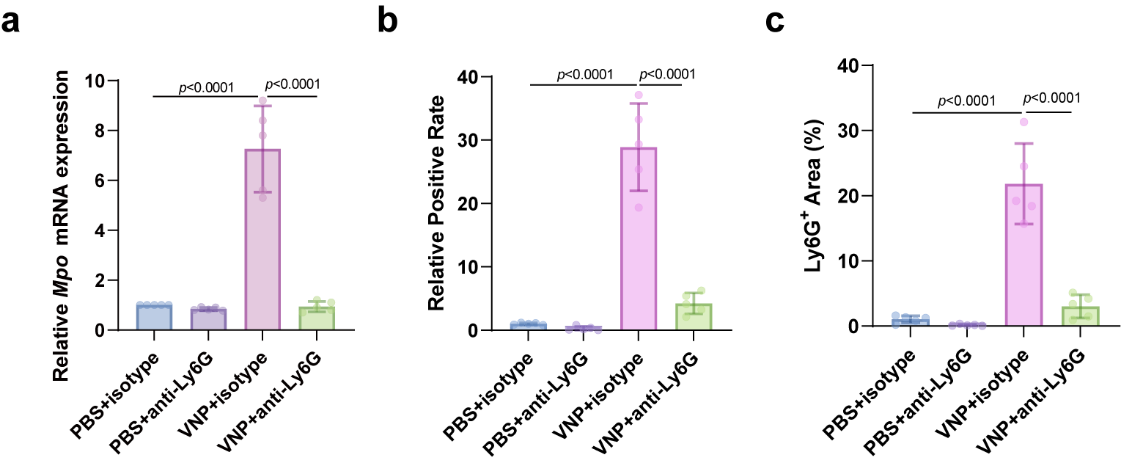


**Figure S5.** VNP combined with neutrophil depletion antibody reduced intratumoral neutrophil infiltration. **a** Relative mRNA expression levels of neutrophil marker genes in tumor tissues. **b-c** Statistical graph of MPO (**b**) and Ly6G (**c**) positive area within tumor. Data represent the mean ± S.D. in a-c (*n* = 5). Statistical significance was determined using one-way ANOVA with Tukey test in a-c.


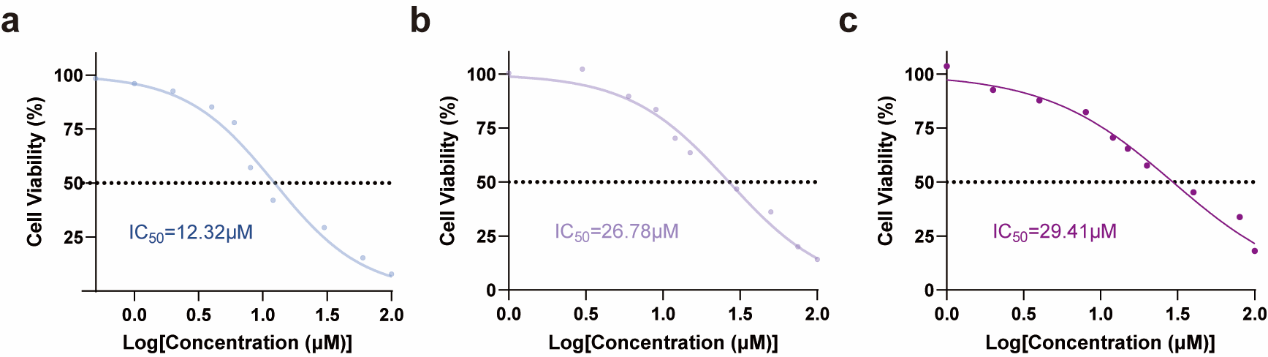


**Figure S6.** The cytotoxic effects of AQ on several cell types were assessed using the CCK-8 assay. **a** B16F10 cell. **b** AML12. **c** NIH3T3 cell.


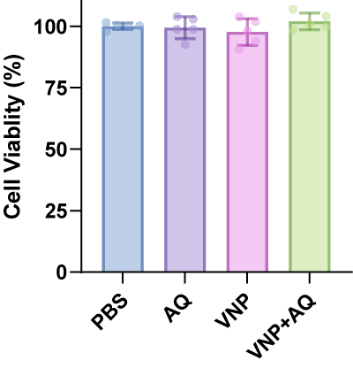


**Figure S7.** Impact of VNP plus AQ combination on murine macrophage viability. Log-phase Raw264.7 cells were seeded at 1 × 10⁴ cells/well in antibiotic-free DMEM, exposed to vehicle, AQ (3 μM), VNP (1 × 10⁵ CFU), or VNP + AQ, and incubated for 12 h (37 °C, 5 % CO₂). Cell viability was then assessed by CCK-8 assay (*n* = 5). Data represent the mean ± S.D. Statistical significance was determined using one-way ANOVA with Tukey test.


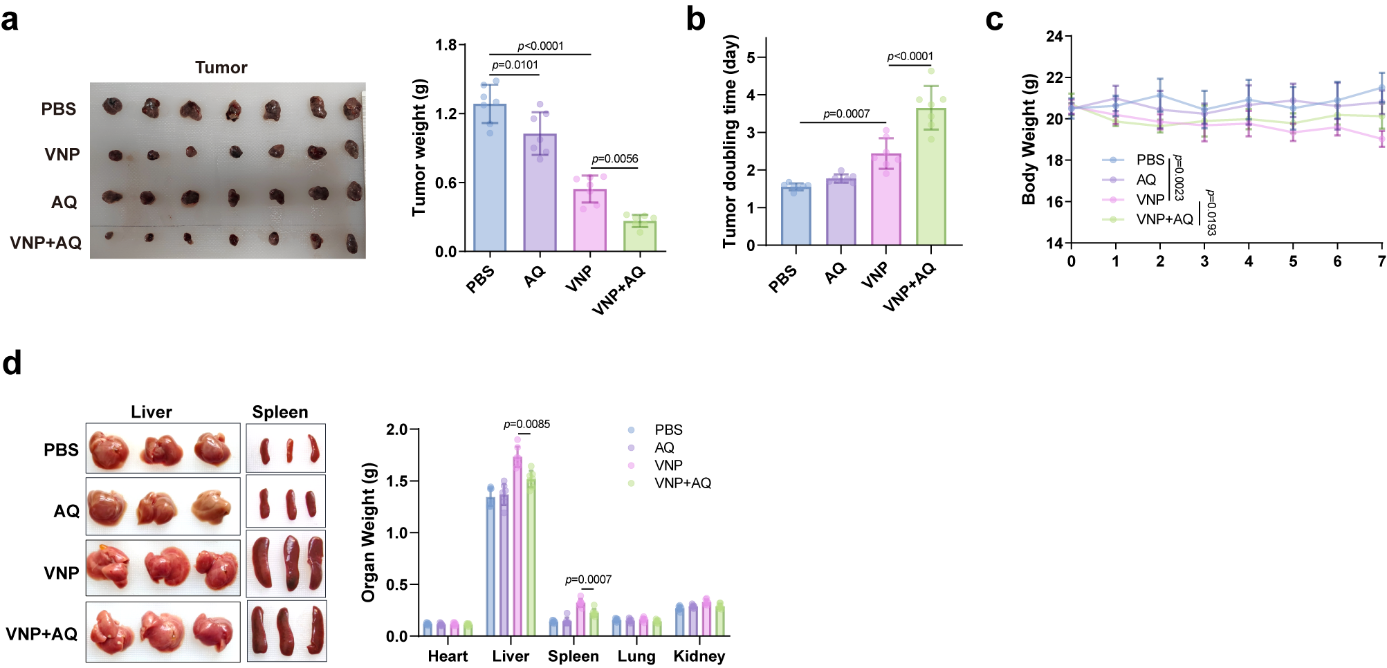


**Figure S8.** VNP combined with AQ enhanced antitumor efficacy. **a** Tumor weight at the end point. **b** Tumor doubling time. **c** Daily body weight changes of mice following VNP treatment. **d** Organ weights. Data represent the mean ± S.D. in a-d (*n* = 7). Statistical significance was determined using one-way ANOVA with Tukey test in a-b. Two-way ANOVA with Tukey test was used in c-d.


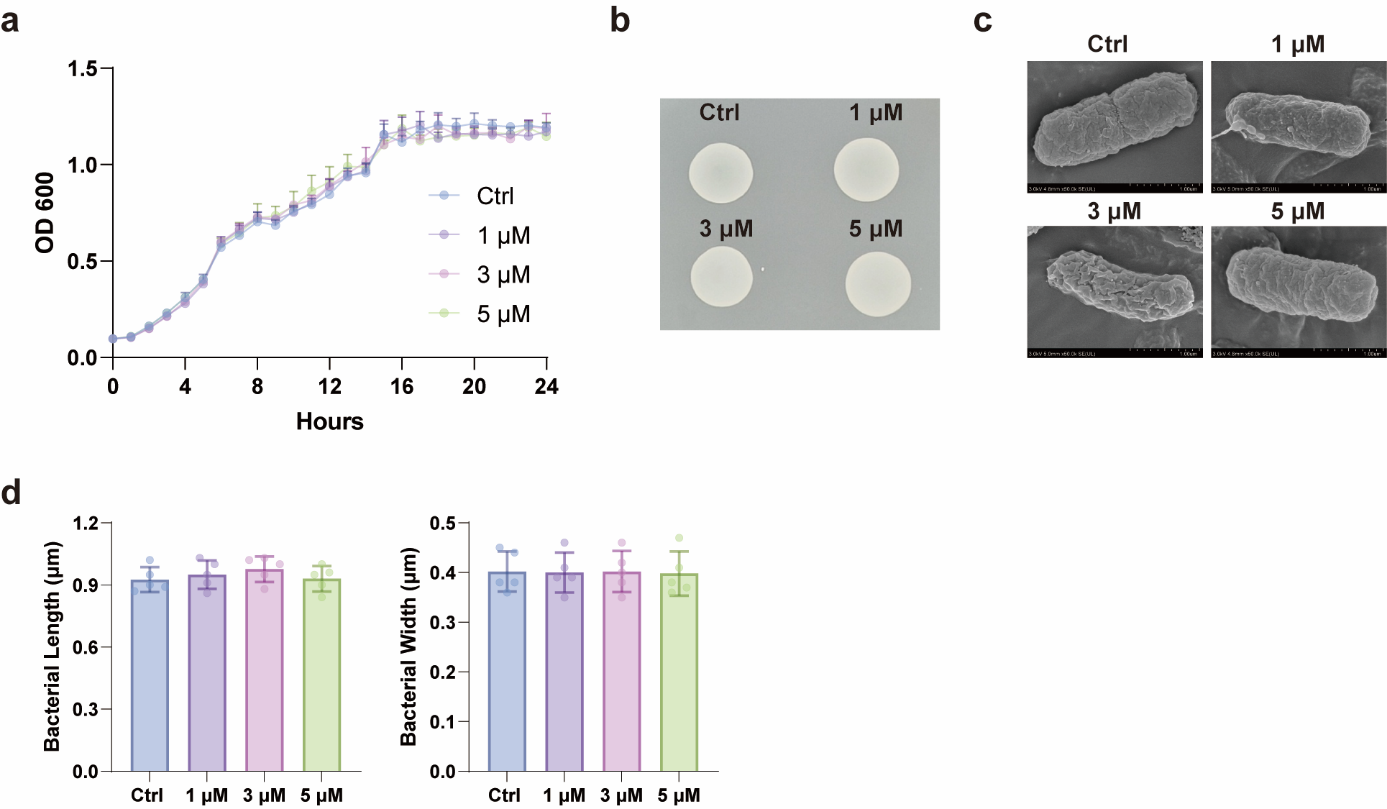


**Figure S9.** AQ leaves the phenotypic signature of VNP unaltered. **a** Time-resolved growth of VNP in LB medium containing escalating AQ concentrations; OD600 recorded at 60-min intervals for 24 h. **b** Colony-forming capacity on LB agar after 24 h AQ exposure. **c** Representative scanning electron micrographs of VNP after 24 h with or without AQ. Scale bar, 1 µm. **d** Quantitative morphometric analysis of bacterial length and width. Data represent the mean ± S.D. in a, d (*n* = 5). Statistical significance was determined using One-way ANOVA with Tukey test in d. Two-way ANOVA with Tukey test was used in a.


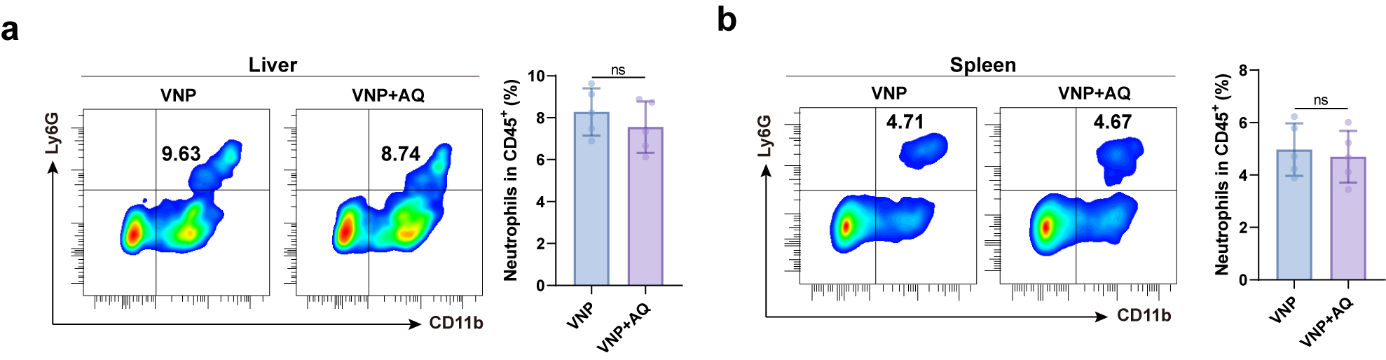


**Figure S10.** The impact of VNP combined with AQ therapy on hepatic and splenic microenvironments of mice. **a**-**b** The proportion of neutrophils among immune cells in the liver (**a**) and spleen (**b**) of mice after combined treatment with VNP and neutrophil neutralizing antibodies. Data represent the mean ± S.D. in a-b (*n* = 5). Statistical significance was determined using unpaired Student’s *t*-test in a-b.


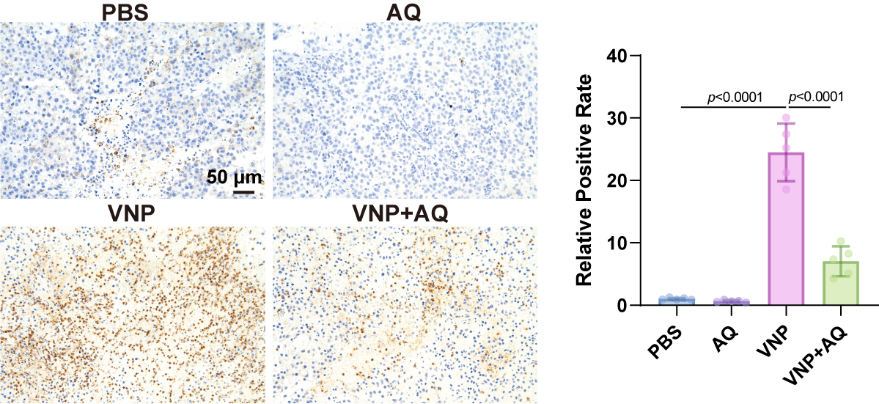


**Figure S11.** VNP combined with AQ reduced intratumoral neutrophil infiltration (*n* = 5). MPO immunohistochemical staining of tumor tissues. Scale bar = 50 μm. Data represent the mean ± S.D. Statistical significance was determined using one-way ANOVA with Tukey test.


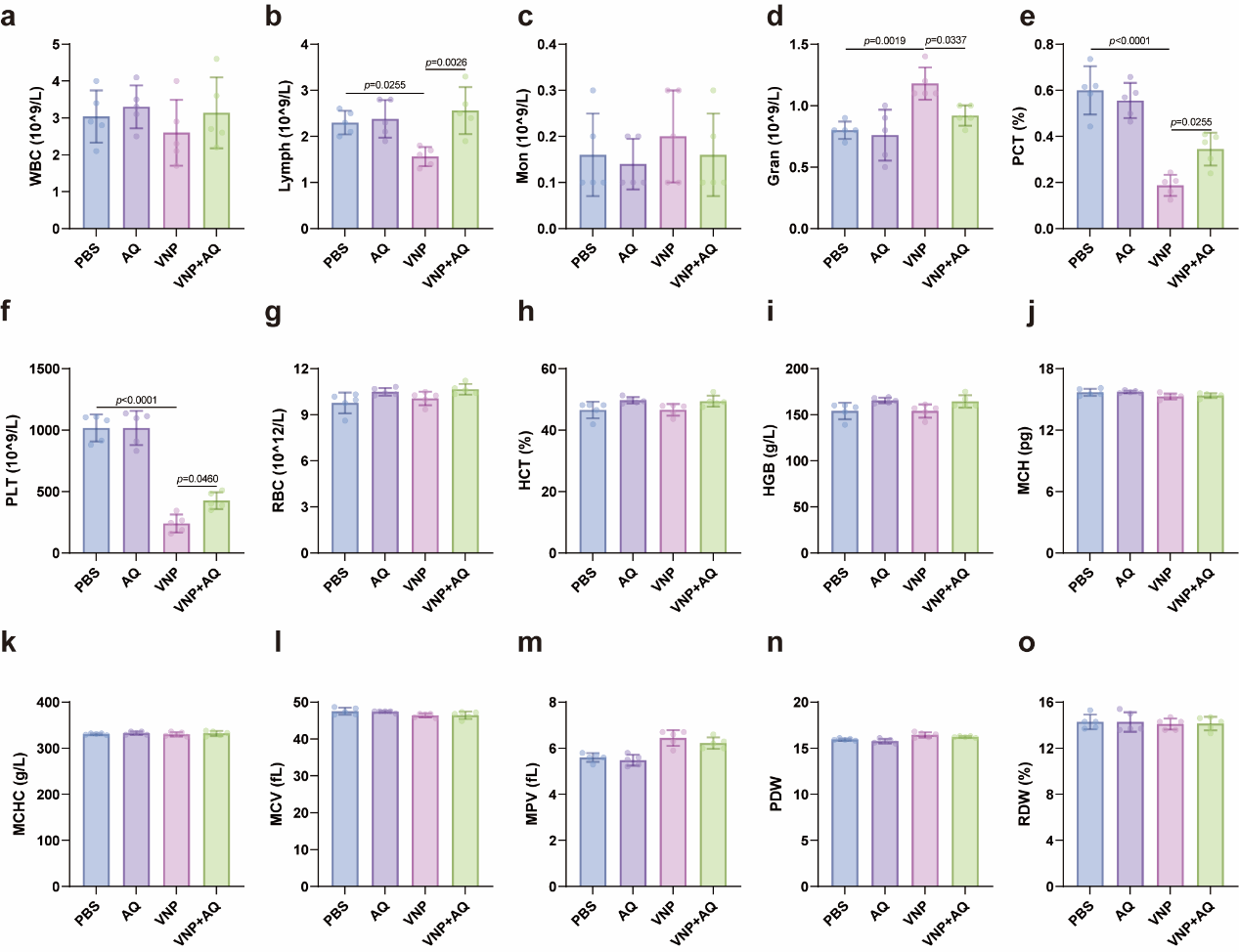


**Figure S12.** Safety assessment of VNP combined with AQ therapy. **a–o** Complete blood count (CBC) analysis in mice. Data represent the mean ± S.D. in a-o (*n* = 5). Statistical significance was determined using one-way ANOVA with Tukey test in a-n.


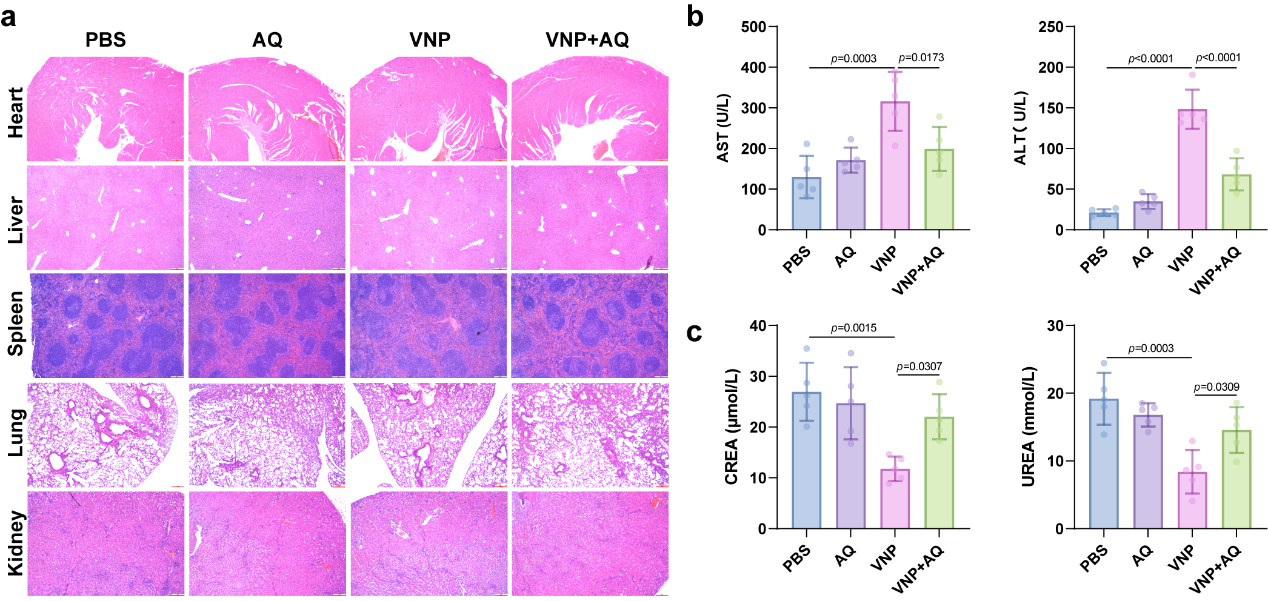


**Figure S13.** Pathological evaluation of VNP combined with AQ therapy. **a** H&E staining of organs. Scale bar = 200 μm. **b–c** Detection of liver and kidney function markers in mouse serum (*n* = 5). Data represent the mean ± S.D. Statistical significance was determined using one-way ANOVA with Tukey test in b-c.


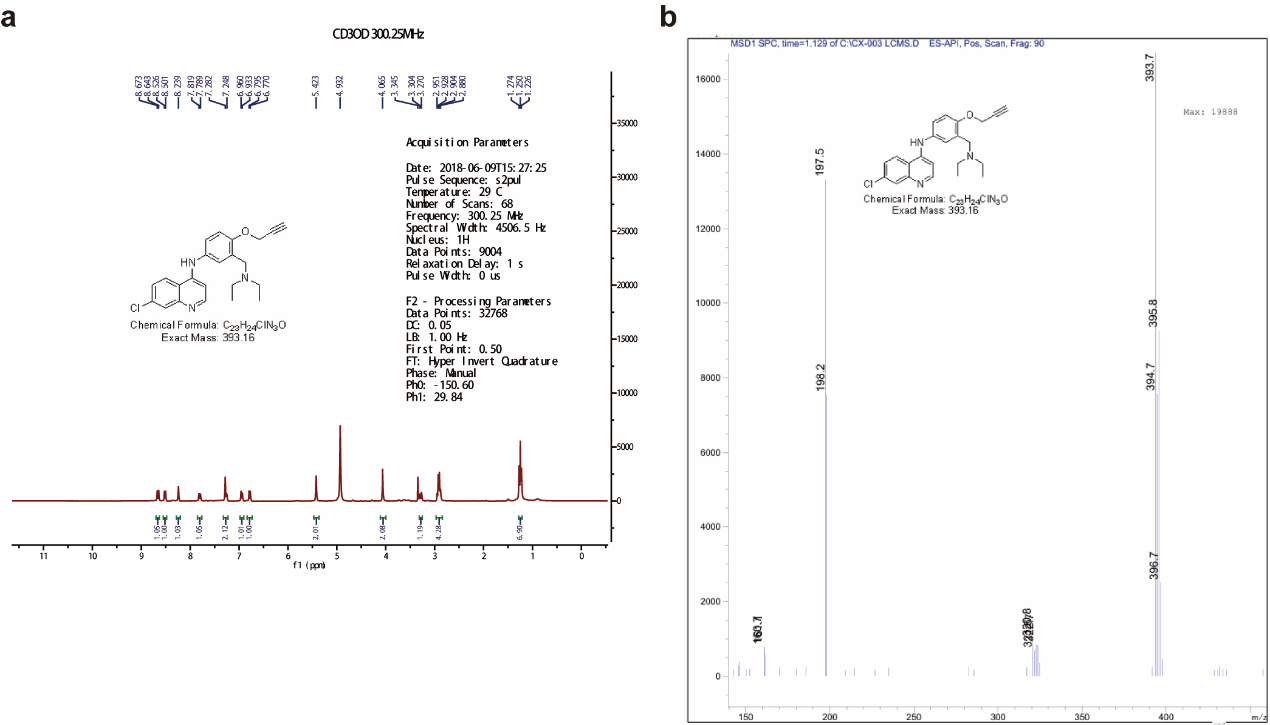


**Figure S14.** The 1H NMR (a) and MS spectra (b) of AQ-P.


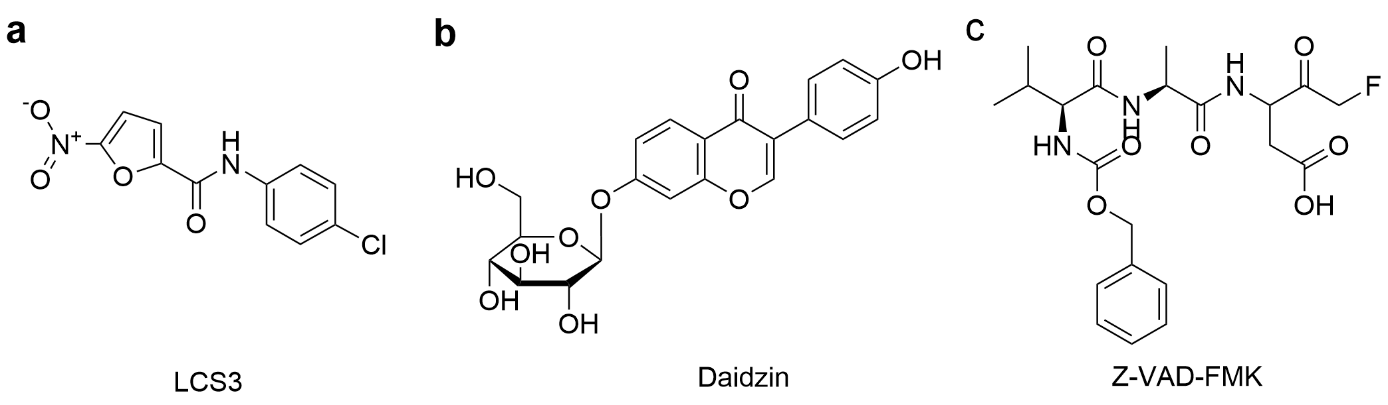


**Figure S15.** Chemical structures of the small-molecule inhibitors. **a-c** Chemical structures of LCS (a), Daidzin (b) and Z-VAD-FMK (c).


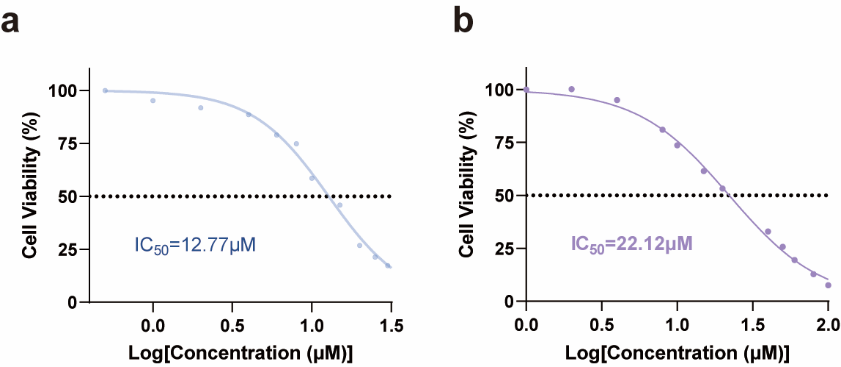


**Figure S16.** The IC_50_ values of inhibitors targeting the candidate target proteins of AQ on neutrophils. **a**-**b** The IC50 values of neutrophils treated with the GR inhibitor LCS3 (**a**) and the ALDH2 inhibitor Daizdin (**b**) (*n* = 5). Data represent the mean ± S.D.


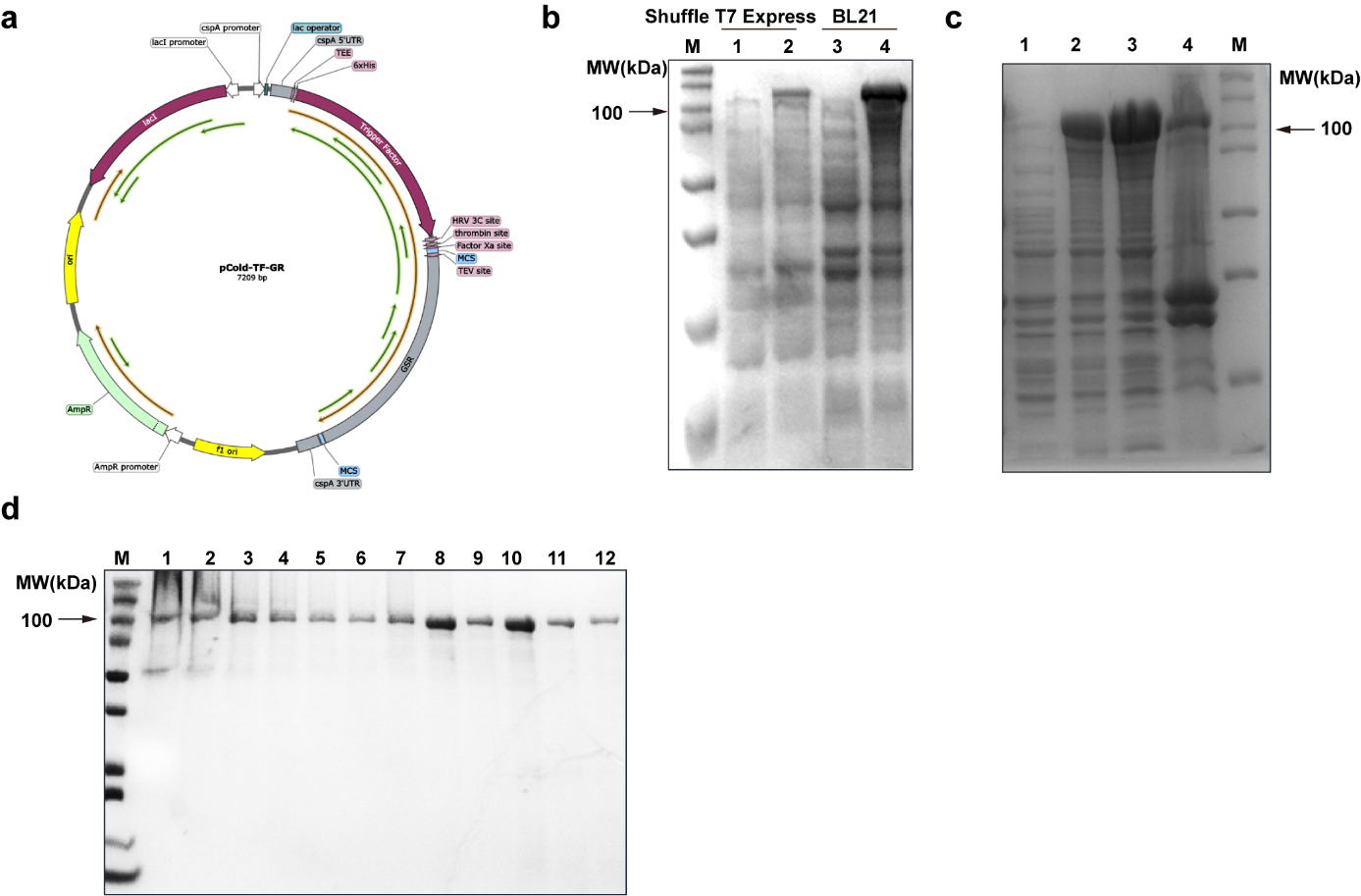


**Figure S17.** Expression and purification of GR protein. **a** Schematic map of the GR expression plasmid. **b** Optimization of GR protein expression vector. M represents the protein marker. Lanes 1 and 2 correspond to samples expressed using the Shuffle T7 Express Lys Y expression vector, with lane 1 representing the uninduced sample and lane 2 representing the sample induced with lactose. Lanes 3 and 4 correspond to samples expressed using the BL21 expression vector, with lane 3 representing the uninduced sample and lane 4 representing the sample induced with lactose. **c** Soluble expression of GR in the bacterial lysate supernatant. Lanes 1 and 2 represent the samples expressed using the BL21 expression vector, with lane 1 corresponding to the uninduced sample and lane 2 to the sample induced with lactose. Lanes 3 and 4 represent the bacterial supernatant and the bacterial pellet obtained after centrifugation post-lysis, respectively. M denotes the protein marker. **d** The GR protein eluted from Ni-affinity chromatography using a 50 mM imidazole elution buffer is represented. Lanes 1–12 correspond to the eluate collected at different time points during the elution process with the 50 mM imidazole buffer.


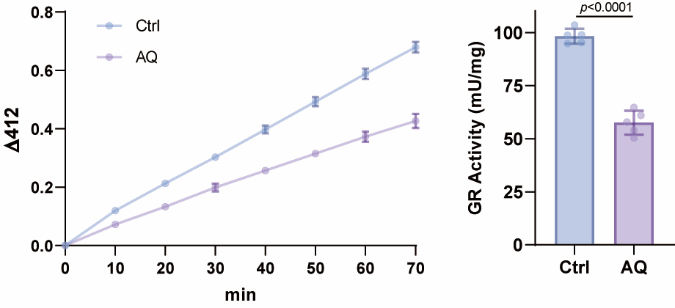


**Figure S18.** The effect of AQ on the enzymatic activity of GR (*n* = 5). Data represent the mean ± S.D. Statistical significance was determined using unpaired Student’s *t*-test.


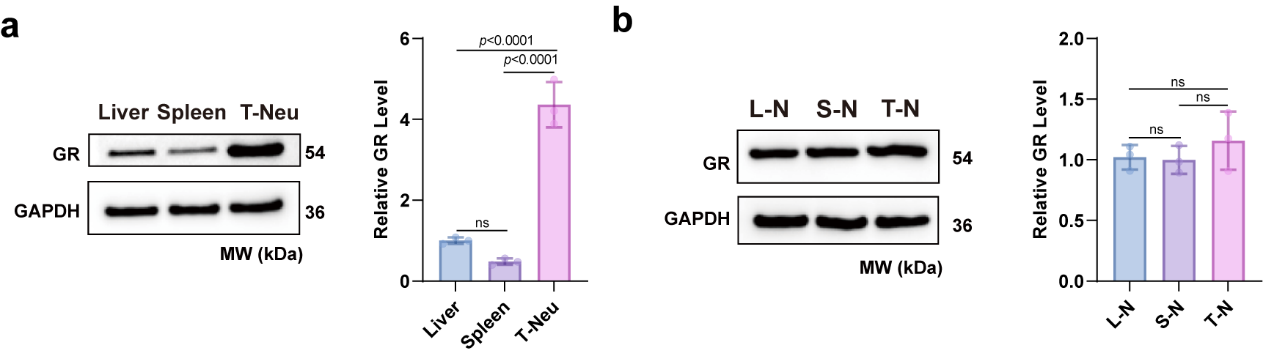


**Figure S19.** GR is highly enriched in neutrophils. **a** Western blot of GR protein in lysates from major organs and from tumour-infiltrating neutrophils harvested after VNP therapy (T-N) (*n* = 3). **b** GR expression in neutrophils purified by anti-Ly6G magnetic beads from liver (L-N), spleen (S-N) and VNP-treated tumours (T-N) (*n* = 3). Data represent the mean ± S.D. Statistical significance was determined using one-way ANOVA with Tukey test in a-b.


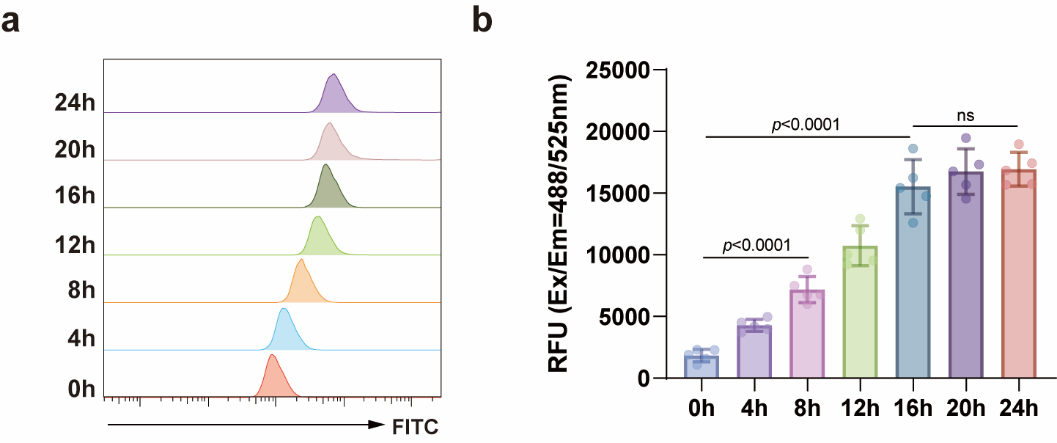


**Figure S20.** AQ elicits a time-dependent escalation of intracellular ROS in neutrophils. **a-b** ROS abundance was quantified by FACS (**a**) and microplate (**b**) after exposure to AQ for the indicated durations (*n* = 5). Data represent the mean ± S.D. Statistical significance was determined using one-way ANOVA with Tukey test in b.


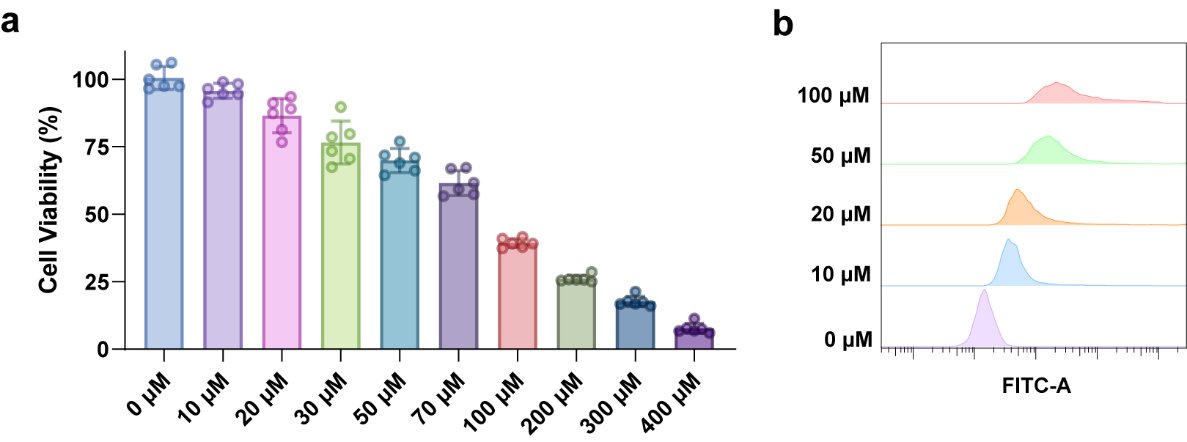


**Figure S21.** The effects of H₂O₂ on neutrophil viability and ROS levels. **a** The impact of H₂O₂ on neutrophil viability was assessed using the CCK-8 assay (*n* = 6). **b** The influence of H₂O₂ on ROS levels in neutrophils was evaluated by flow cytometry. Data represent the mean ± S.D. Statistical significance was determined using one-way ANOVA with Tukey test in a.


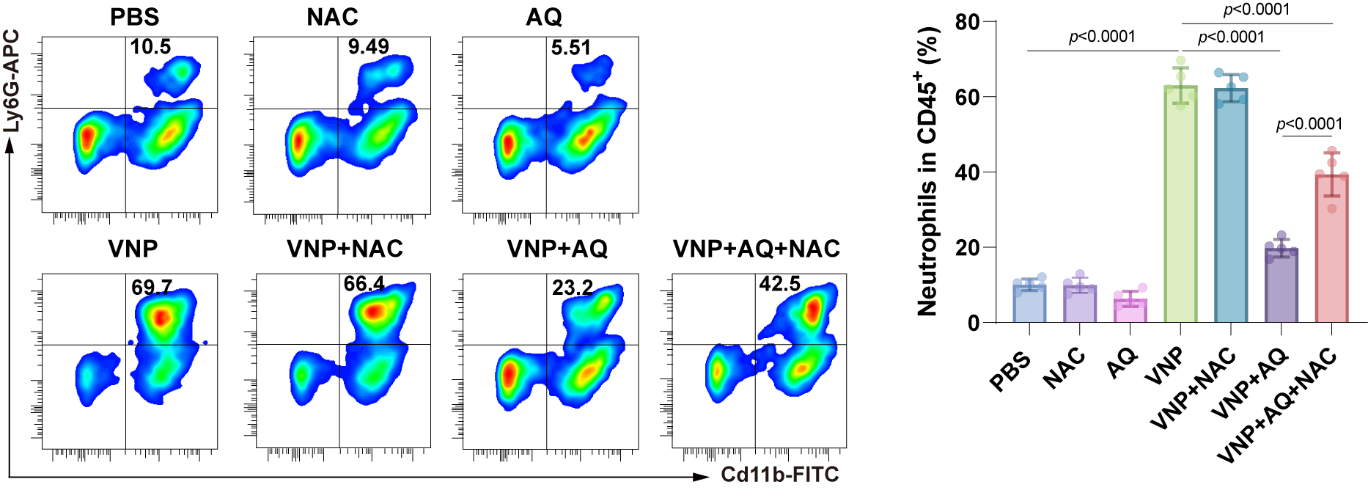


**Figure S22.** N-acetylcysteine restores AQ-elicited depletion of tumour-associated neutrophils through scavenging of the intratumoral ROS burst (*n* = 5). Data represent the mean ± S.D. Statistical significance was determined using one-way ANOVA with Tukey test.


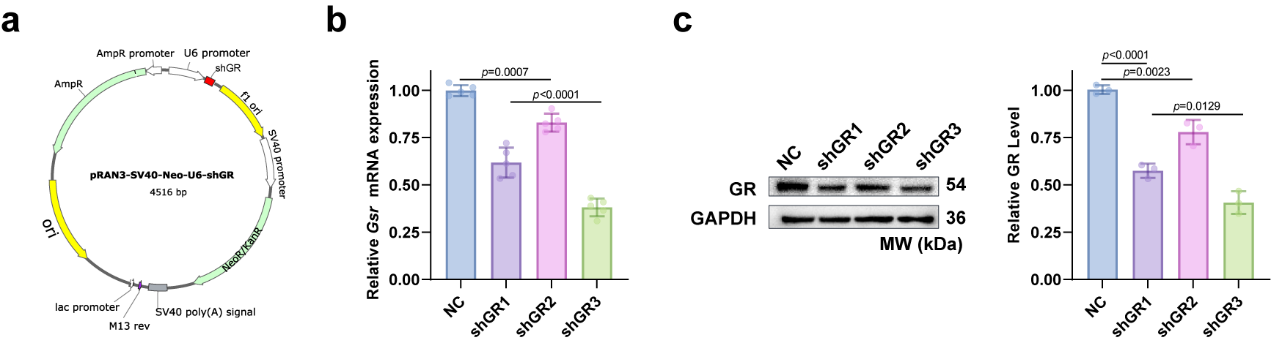


**Figure S23.** shGR3 exhibited superior knockdown efficiency. **a** Schematic map of the shGR plasmid. **b–c** Validation of the interference efficiency of the plasmid in B16F10 cells by quantitative PCR (**b**) and Western blot (**c**). B16F10 cells were seeded in 12-well plates and transfected at 70–80 % confluence with 1 µg shGR plasmid that had been pre-complexed with Lipofectamine 2000 (1:2 DNA-to-reagent volume ratio) following a 30-min room-temperature incubation; knock-down efficiency was quantified 36 h later. Data represent the mean ± S.D. in b (*n* = 5) and c (*n* = 3). Statistical significance was determined using one-way ANOVA with Tukey test in b-c.


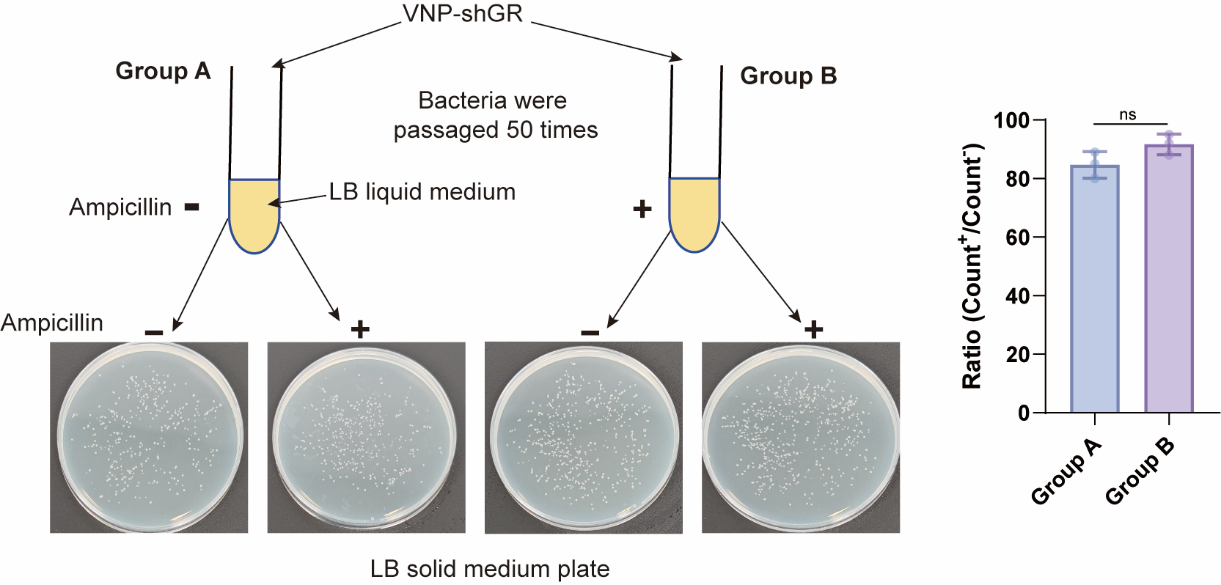


**Figure S24.** Stability of the shGR-bearing plasmid in VNP. Mid-log-phase VNP-shGR was inoculated 1:5 into LB broth without (group A) or with ampicillin (group B) and cultivated at 37 °C, 200 rpm for 4 h. Both groups were then passaged 50 times (4-h intervals). Equal OD-adjusted volumes from groups A and B were plated on LB agar ± ampicillin, incubated inverted at 37 °C for 16 h, and enumerated; the resulting CFU ratios are shown on the right (*n* = 3). Data represent the mean ± S.D. Statistical significance was determined using unpaired Student’s *t*-test.


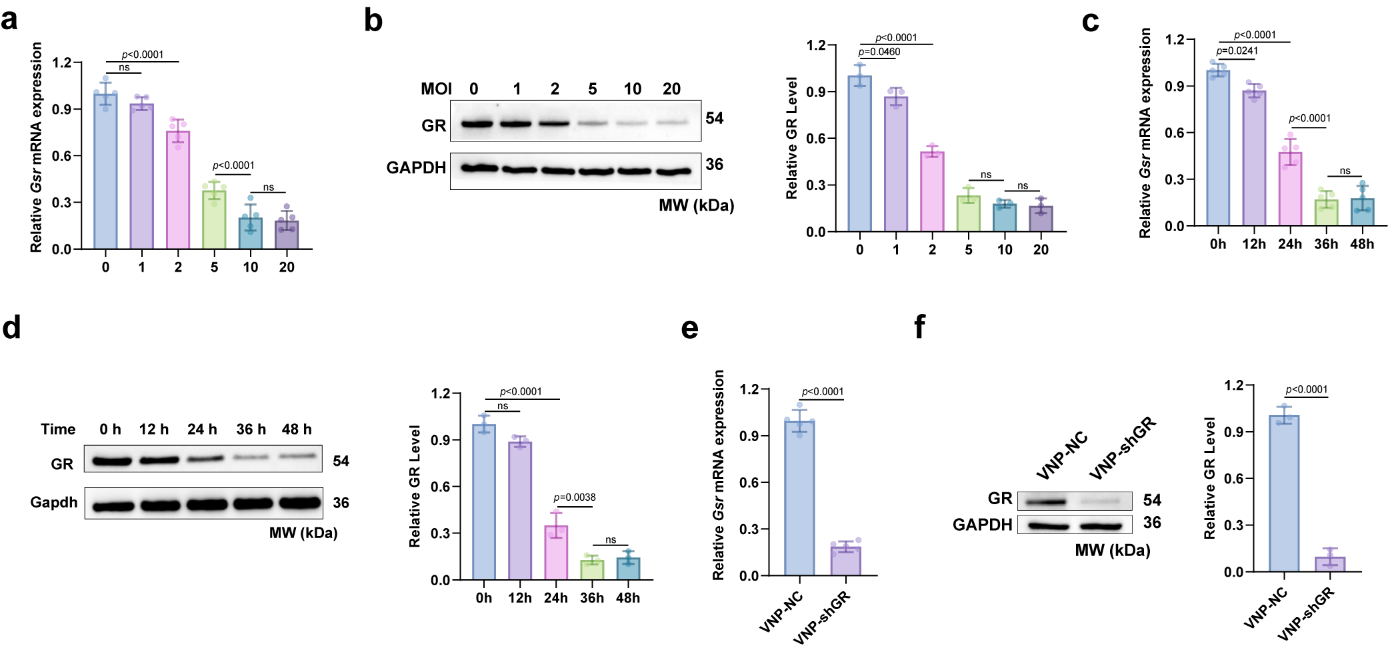


**Figure S25.** Maximal GR silencing in neutrophils was obtained by exposing the cells to VNP-shGR at an MOI of 10 for 36 h. **a**-**b** GR mRNA (**a**) and protein (**b**) levels in bone-marrow-derived neutrophils (5 × 10⁵ cells) infected for 36 h with VNP-shGR at the indicated MOI. **c-d** Time-resolved expression of GR mRNA (**c**) and protein (**d**) in neutrophils (5 × 10⁵) challenged with VNP-shGR (MOI = 10). **e-f** Validation of VNP-shGR3 knock-down efficiency in neutrophils. Bone-marrow-derived neutrophils (5 × 10⁵) were infected with VNP-shGR at MOI 10 for 36 h, after which GR mRNA (**e**) and protein (**f**) were quantified. Data represent the mean ± S.D. in a, c, e (*n* = 5) and b, d, f (*n* = 3). Statistical significance was determined using unpaired Student’s *t*-test in e-f. One-way ANOVA with Tukey test was used in a-d.


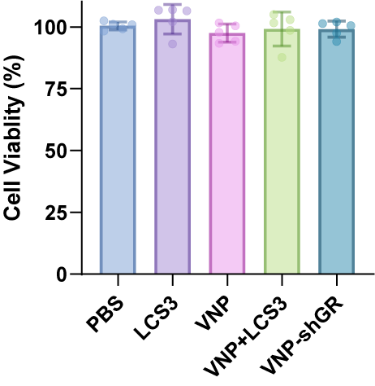


**Figure S26.** Impact of VNP-shGR therapy on murine macrophage viability. Log-phase Raw264.7 cells were seeded at 1 × 10⁴ cells/well in antibiotic-free DMEM, exposed to vehicle, LCS (5 μM), VNP (1 × 10⁵ CFU), VNP + LCS3 and VNP-shGR (1 × 10⁵ CFU), and incubated for 12 h (37 °C, 5 % CO₂). Cell viability was then assessed by CCK-8 assay (*n* = 5). Data represent the mean ± S.D. Statistical significance was determined using one-way ANOVA with Tukey test.


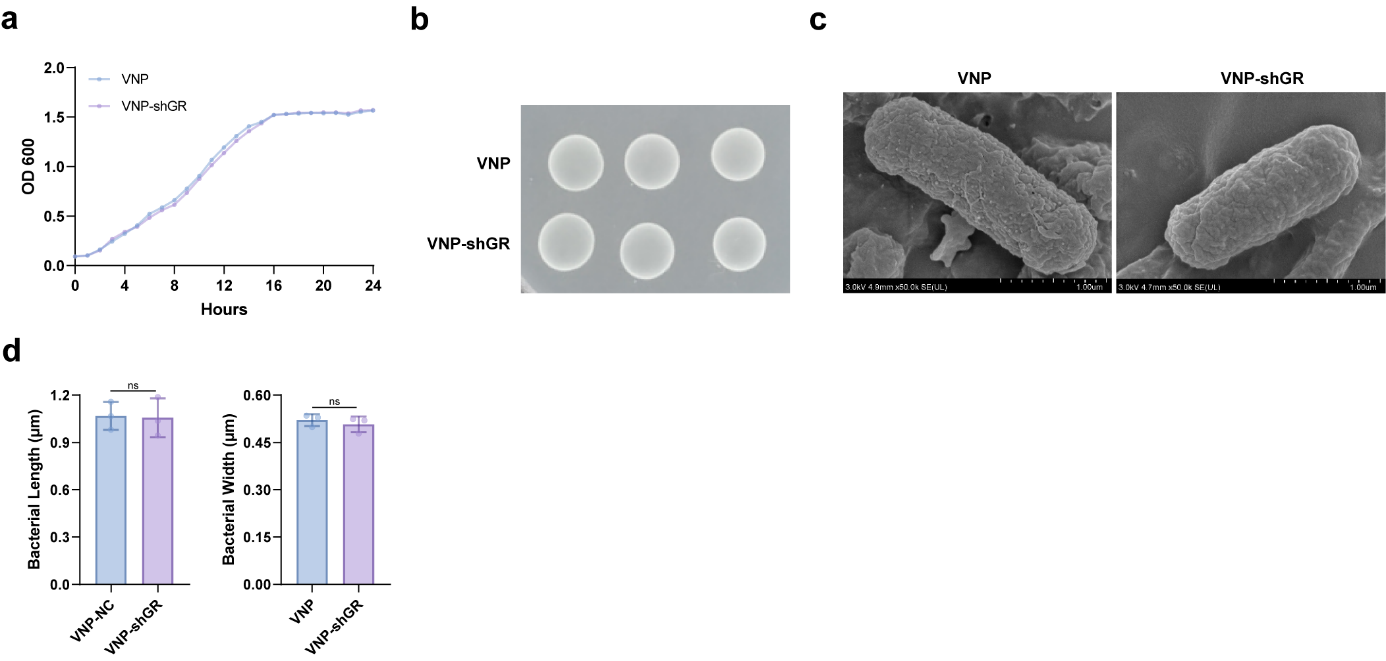


**Figure S27.** Characterization of VNP-shGR. **a** Growth curve of VNP-shGR. **b** Colony-forming efficiency of VNP and VNP-shGR on LB agar after 16 h incubation. **c** Morphological observation of VNP-shGR using scanning electron microscopy. Scale bar =1 μm. **d** Quantitative morphometric analysis of bacterial length and width. Data represent the mean ± S.D. in a (*n* = 5) and d (*n* = 3). Statistical significance was determined using unpaired Student’s *t*-test in d. Two-way ANOVA with Tukey test was used in a.


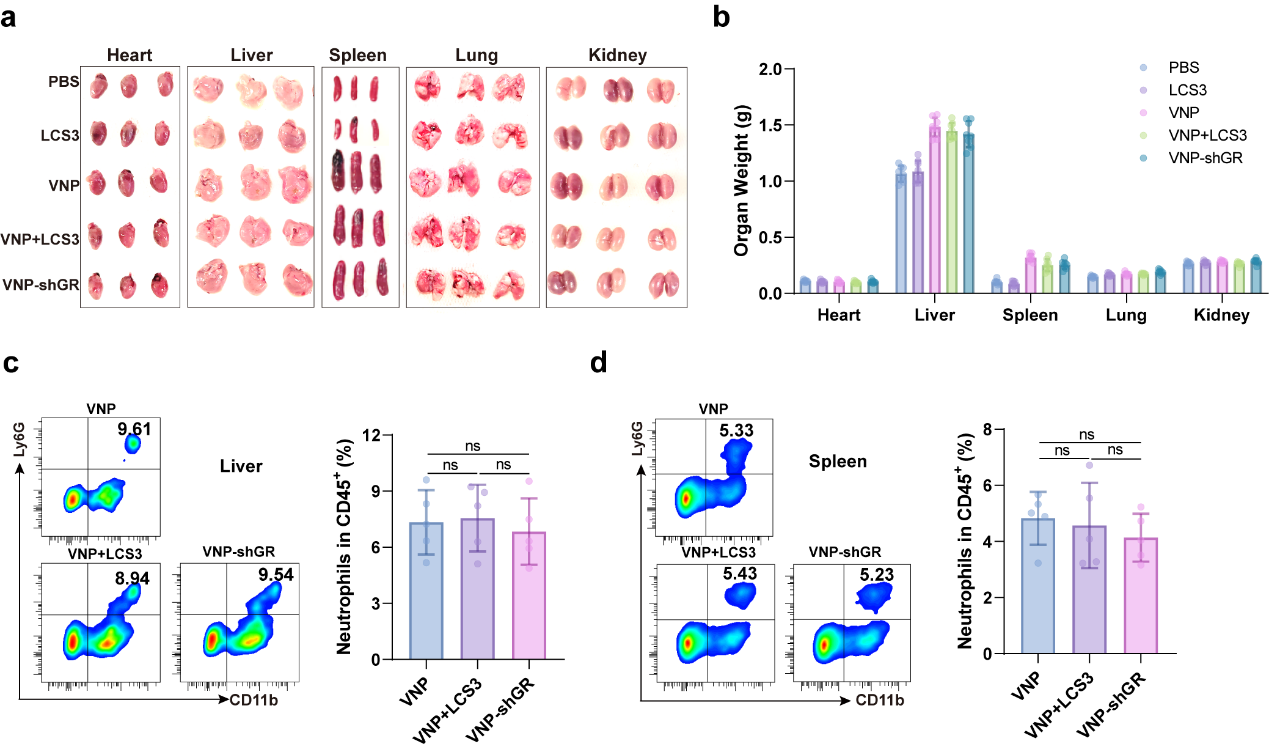


**Figure S28.** The impact of VNP-shGR and VNP combined with GR inhibitor therapy on hepatic and splenic microenvironments of mice. **a** Representative images of mouse heart, liver, spleen, lungs, and kidneys. **b** Organ weights. **c**-**d**. The proportion of neutrophils among immune cells in the liver (**c**) and spleen (**d**) of mice after VNP-shGR and VNP combined with GR inhibitor therapy. Data represent the mean ± S.D. in a (*n* = 3), b (*n* = 8), c-d (*n* = 5). Statistical significance was determined using two-way ANOVA with Tukey test in b. One-way ANOVA with Tukey test was used in c-d.


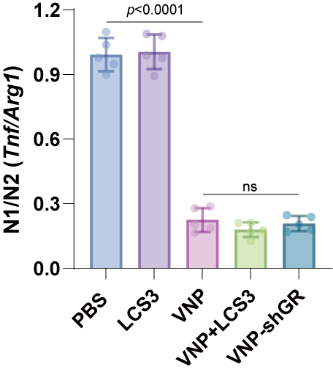


**Figure S29.** Impact of VNP-shGR on intratumoral N1/N2 neutrophil ratios (*n* = 5). Data represent the mean ± S.D. Statistical significance was determined using one-way ANOVA with Tukey test.


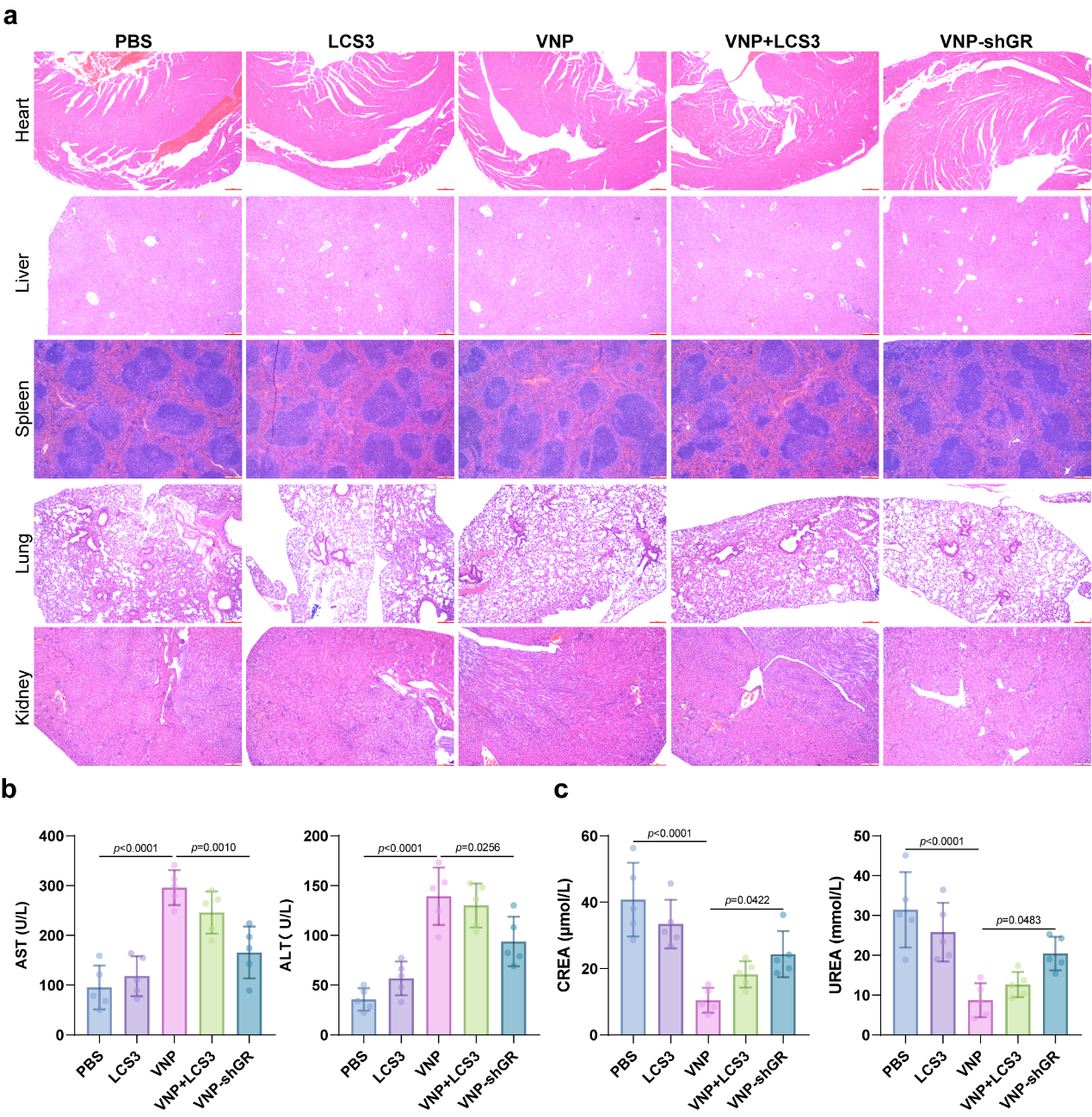


**Figure S30.** Safety assessment of VNP-shGR therapy. **a** H&E staining of organs. Scale bar =200 μm. **b–c** Detection of liver and kidney function markers in mouse serum (*n* = 5). Data represent the mean ± S.D. Statistical significance was determined using one-way ANOVA with Tukey test in b-c.


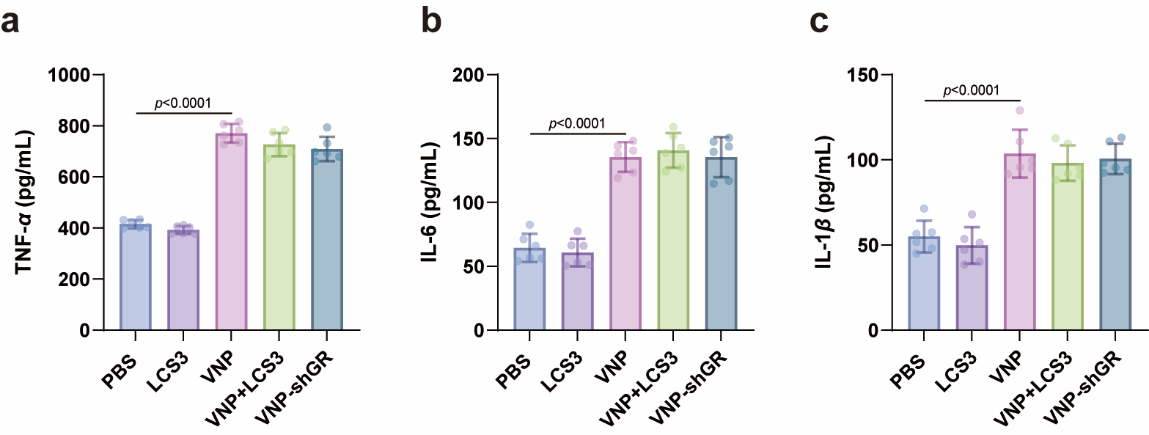


**Figure S31.** Serum inflammatory cytokine (TNF-α, IL-6 and IL-1β) levels in the indicated groups were determined by ELISA (*n* = 6). Data represent the mean ± S.D. Statistical significance was determined using one-way ANOVA with Tukey test.


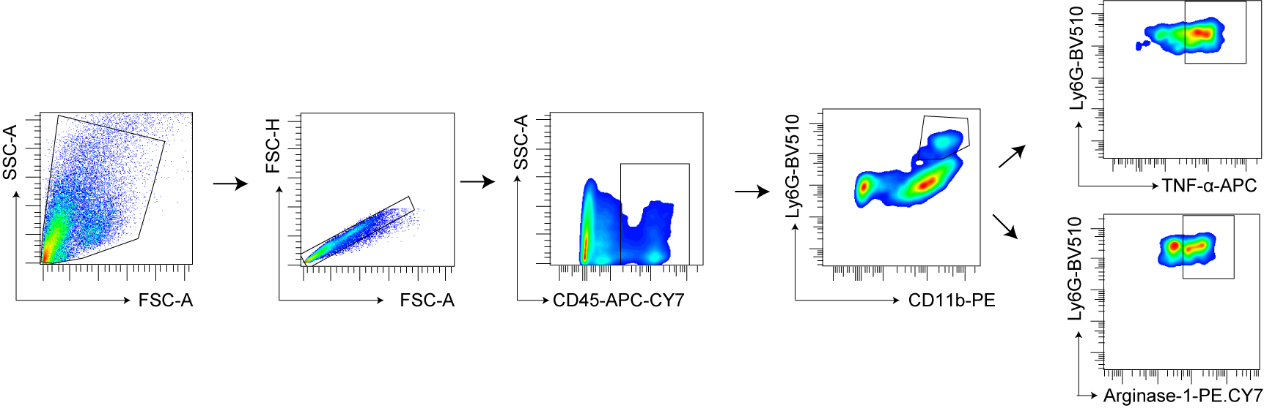


**Figure S32.** Gating strategy for FACS. Single-cell suspensions were sequentially gated to exclude debris (FSC-A vs. SSC-A) and doublets (FSC-H vs. FSC-A), followed by selection of CD45⁺ live leukocytes. Neutrophils were identified as CD11b⁺Ly6G⁺ within this population, and after fixation and permeabilisation were phenotyped as TNF-α⁺ (N1) or Arginase-1⁺ (N2).

**Table S1. The primer sequence of RT-PCR.**

| **Primer** | **Forward (5'-3')** | **Reverse（5'-3'）** |
| --- | --- | --- |
| **GR** | **CACGGCTATGCAACATTCGC** | **GTGTGGAGCGGTAAACTTTTTC** |
| **MMP-9** | **GCGTCATTCGCGTGGATAAG** | **TGGAAACTCACACGCCAGAA** |
| **TNF-α** | **GCCACGGCACAGTCATTGA** | **TGCTGATGGCCTGATTGTCTT** |
| **CD86** | **CTGCTCTCTGCTAACTTCAGTCAAC** | **GTTCTGGGTAACCGTGTATAGATGAG** |
| **β-Actin** | **ACTCTTCCAGCCTTCCTTCCT** | **CAGTGATCTCCTTCTGCATCC** |
| **ELANE** | **AGCAGTCCATTGTGTGAACGG** | **CACAGCCTCCTCGGATGAAG** |
| **MPO** | **GCTCCTTGCCTGCCTCATTGG** | **TGCCAGTGTTGTCACAGATGATACG** |
| **Fas** | **TTTAAAGCTGAGGAGGCGGG** | **TCAGGTTGGCATGGTTGACA** |
| **Ccl-2** | **CAGCCAGATGCAGTTAACGC** | **GCTGCTGGTGATCCTCTTGT** |
| **ICAM-1** | **GGACCACGGAGCCAATTTC** | **CTCGGAGACATTAGAGAACAATGC** |
| **TGF-β** | **CTTTGTACAACAGCACCCGC** | **CATAGATGGCGTTGTTGCGG** |
| **Arginase-1** | **AAGATTCCCGATGTGCCAGG** | **GTCCACGTCTCTCAAGCCAA** |
| **PD-L1** | **CAGGACGCAGGCGTTTACTG** | **CTTCCCACTCACGGGTTGGT** |
| **VEGF-α** | **CCACGACAGAAGGAGAGCAGAAG** | **ACAGGACGGCTTGAAGATGTACTC** |

**Table S2. The sequence of shGR.**

| **Names** | **Sequence（5’-3’）** |
| --- | --- |
| **shGR-1-Forward** | **gatccGCTGTTCATAAGTATGGGAAActcgagTTTCCCATACTTATGAACAGCtttttt** |
| **shGR-1-Reverse** | **aattaaaaaaGCTGTTCATAAGTATGGGAAActcgagTTTCCCATACTTATGAACAGCg** |
| **shGR-2-Forward** | **gatccgCGCCTGAACACCATCTATCAActcgagTTGATAGATGGTGTTCAGGCGtttttt** |
| **shGR-2-Reverse** | **aattaaaaaaCGCCTGAACACCATCTATCAActcgagTTGATAGATGGTGTTCAGGCGcg** |
| **shGR-3-Forward** | **gatccGTGTTGAAGTTCACACAGGTTctcgagAACCTGTGTGAACTTCAACACtttttt** |
| **shGR-3-Reverse** | **aattaaaaaaGTGTTGAAGTTCACACAGGTTctcgagAACCTGTGTGAACTTCAACACg** |

**Table S3. List of target proteins of AQ from ABPP**

| Accession | Name | Species | Peptides (95%) |
| --- | --- | --- | --- |
| sp\|P47791\|GSHR_MOUSE | Glutathione reductase, mitochondrial OS=Mus musculus OX=10090 GN=Gsr PE=1 SV=3 | Mouse | 2 |
| tr\|Q544B1\|Q544B1_MOUSE | Aldehyde dehydrogenase 2, mitochondrial, isoform CRA_b OS=Mus musculus OX=10090 GN=Aldh2 PE=1 SV=1 | Mouse | 1 |
| tr\|Q6DFY1\|Q6DFY1_MOUSE | Gda protein OS=Mus musculus OX=10090 GN=Gda PE=2 SV=1 | Mouse | 3 |
| tr\|Q58E64\|Q58E64_MOUSE | Elongation factor 1-alpha OS=Mus musculus OX=10090 GN=Eef1a1 PE=1 SV=1 | Mouse | 2 |
| sp\|P62631\|spEF1A2_MOUSE | Elongation factor 1-alpha 2 OS=Mus musculus OX=10090 GN=Eef1a2 PE=1 SV=1 | Mouse | 1 |
| tr\|Q4FZL1\|Q4FZL1_MOUSE | Eif4a1 protein (Fragment) OS=Mus musculus OX=10090 GN=Eif4a1 PE=2 SV=1 | Mouse | 2 |
| tr\|B2RVZ3\|B2RVZ3_MOUSE | Guanine nucleotide binding protein, alpha transducing 3 OS=Mus musculus OX=10090 GN=Gnat3 PE=2 SV=1 | Mouse | 1 |
| sp\|P27773\|PDIA3_MOUSE | Protein disulfide-isomerase A3 OS=Mus musculus OX=10090 GN=Pdia3 PE=1 SV=2 | Mouse | 1 |
